# Process intensification of the ionoSolv pretreatment: effects of biomass loading, particle size and scale-up from 10 mL to 1 L

**DOI:** 10.1038/s41598-021-94629-z

**Published:** 2021-07-28

**Authors:** Clementine L. Chambon, Pedro Verdía, Paul S. Fennell, Jason P. Hallett

**Affiliations:** Department of Chemical Engineering, Imperial College London, Exhibition Road, South Kensington, London, SW7 2AZ UK

**Keywords:** Chemical engineering, Bioalcohols, Ionic liquids

## Abstract

The ionoSolv process is one of the most promising technologies for biomass pretreatment in a biorefinery context. In order to evaluate the transition of the ionoSolv pretreatment of biomass from bench-scale experiments to commercial scale, there is a need to get better insight in process intensification. In this work, the effects of biomass loading, particle size, pulp washing protocols and 100-fold scale up for the pretreatment of the grassy biomass *Miscanthus giganteus* with the IL triethylammonium hydrogen sulfate, [TEA][HSO_4_], are presented as a necessary step in that direction. At the bench scale, increasing biomass loading from 10 to 50 wt% reduced glucose yields from 68 to 23% due to re-precipitation of lignin onto the pulp surface. Omitting the pulp air-drying step maintained saccharification yields at 66% at 50 wt% loading due to reduced fiber hornification. 100-fold scale-up (from 10 mL to 1 L) improved the efficacy of ionoSolv pretreatment and increasing loadings from 10 to 20 wt% reduced lignin reprecipitation and led to higher glucose yields due to the improved heat and mass transfer caused by efficient slurry mixing in the reactor. Pretreatment of particle sizes of 1–3 mm was more effective than fine powders (0.18–0.85 mm) giving higher glucose yields due to reduced surface area available for lignin re-precipitation while reducing grinding energy needs. Stirred ionoSolv pretreatment showed great potential for industrialization and further process intensification after optimization of the pretreatment conditions (temperature, residence time, stirring speed), particle size and biomass loading. Pulp washing protocols need further improvement to reduce the incidence of lignin precipitation and the water requirements of lignin washing.

## Introduction

Chemical pretreatment is an essential step in the conversion of lignocellulosic materials to biofuels, chemicals and other products. It is considered the second most expensive contributor to conversion expenses, after feedstock (~ 30%), representing ~ 14%^1^. Therefore, there is a need to reduce its associated costs. One of the most promising biomass pretreatment methods is the IonoSolv process, which utilizes low cost protic ionic liquids (PILs) as pretreatment media^2,3^. As opposed to other IL-based pretreatment processes, the IonoSolv process dissolves the lignin and hemicellulose fractions of the biomass, that can be recovered and valorized separately, leaving a cellulose-rich pulp as solid residue. Our previous work has demonstrated that hardwood, softwood and grassy biomass feedstocks can be deconstructed and fractionated with PILs, producing a highly digestible cellulose-rich pulp and lignin with tunable properties^3–5^. The alkylammonium-based ILs used have high thermal stability, can be produced on the bulk at price ranges around 0.78 $/kg^6^, and could be recycled multiple times, according to studies conducted at the bench scale (10–20 mL batch size without stirring)^4^. The ionic liquid (IL) cost, IL-to-biomass ratio (i.e. solids loading) and the IL recycling rate have been identified as focus areas for more cost-effective pretreatments^7^.

Increasing solid loadings could help lowering capital costs and energy requirements for heating and cooling because reactor size and solvent cost trend approximately inversely with it^7–10^. Doubling the solids loading can be expected to decrease reactor CAPEX by approximately 40%, making this parameter essential to improve economic viability of pretreatments processes^11^. However, high solids concentrations increase the viscosity of the biomass-IL slurry^12^, introducing heat and mass transfer limitations and higher power requirements for stirring^7,13,14^. Most pretreatment studies at the bench scale are performed with solids loadings between 5–20 wt% where pretreatment efficiency is high. Process intensification studies have mostly examined cellulose-dissolving ILs such as [Emim][OAc], finding trends of decreasing lignin removal efficiency and solid recoveries during switchgrass pretreatment^12^. Saccharification yields decreased significantly above 20 wt% loading, beyond which point the IL was only able to wet the surface of the biomass, ceasing to act as a true solvent^12,15^.

IonoSolv processes are based on the dissolution of lignin and hemicelluloses (20–30 wt% of biomass) but *not* of cellulose (the remaining 60–70 wt%)^3,4^. Viscosity is therefore less dependent on solids loading than for cellulose-dissolving ILs such as [Emim][OAc]^12^. Gschwend et al*.* increased the solid to solvent ratio up to 50 wt% for pretreatment of pine using [DMBA][HSO_4_] at bench scale (~ 10 mL), obtaining glucose yields of 75% after saccharification, compared to quantitative glucose release at 10 wt% loading^11^. It is worthy to note that dissolution of lignin during ionoSolv treatment introduces the possibility of lignin re-depositing onto the cellulose surface^16–18^. This raises the need to adapt pulp washing protocols to avoid lignin re-precipitation and hornification, both of which negatively affect enzymatic hydrolysis yields.

Biomass grinding (comminution) is a highly energy-intensive operation routinely conducted as a first step in many bioconversion processes for transport and processing purposes^19–22^. It has been estimated to account for up to 60% of the total energy of chemical pretreatment^23^ and 10% of the process cost^24^. Hence, developing technologies capable of converting biomass with larger particle sizes is highly desirable and has been investigated using leading pretreatment technologies^25–28^. Larger particles lead to slower penetration of IL into biomass and slower dissolution of biomass components, but also reduce viscosity of the slurry and power requirements for stirring^29,30^.

Dilute acid and alkaline treatments have been found to perform best at a maximal particle size of 3 mm, whereas liquid hot water (15 mm) and steam explosion (50 mm) offer more flexibility for pretreating large particle sizes^23,26^. Studies of cellulose-dissolving ILs were found to maintain high glucose yields using feedstock size of up to 5 mm for rice straw, 6 mm for woody biomass, and 12.5 mm for corn stover^28,31–35^. Among lignin-dissolving ILs, cholinium lysinate, [Ch][Lys], showed high delignification ability for corn stover up to 10 mm at solids loading of 50 wt%^31,33^. Hardwoods and softwoods, which have higher density and lignin content, are significantly more energy-intensive to grind and are expected to be more sensitive to particle size than grassy feedstocks^21,23,26,36^.

So far, the scale-up of IL pretreatment processes has been focused on cellulose-dissolving ILs^37,38^. Pretreatment of switchgrass was scaled up 600-fold (6 L vs 0.01 L) by Li et al^37^ using 15 wt% biomass loading, maintaining high glucose release (95%), similar pulp recoveries and higher delignification. The main challenge was removing residual IL from the recovered pulp, requiring excessive water and water–ethanol washes, as IL can act as an inhibitor to saccharification enzymes and yeasts in fermentation downstream^37,39,40^. With hardwood and mixed feedstocks (hardwood/grass) a 30-fold scale-up to 6 L gave less effective lignin and hemicellulose removal due to the combined effects of scale-up, loading (10 wt%) and temperature severity. However, uniform mixing and more effective biomass washing and recovery by centrifugation increased the recovery of structural carbohydrates^41^.

To date, no scale-up studies of the ionoSolv process have yet been published, yet this process offers several advantages over cellulose-dissolving IL technologies. It uses IL-water mixtures of cheap ILs synthesized from low-cost amines and sulfuric acid at a price competitive with common organic solvents^6^. These solvents have low viscosity, which facilitates handling, and are tolerant to feedstock moisture^42,43^. In our previous works, the robustness of the ionoSolv pretreatment process to varying temperatures, IL cations, solids loadings and feedstocks has been verified over a wide range of conditions^2,42–44^. However, the data obtained at the bench scale cannot be directly translated to industrially-relevant scales. Recent research has shown that even a small scale up in pretreatment from 15 to 100 mL tubes can have a huge impact in the pretreatment outcomes due to different heating profiles, container geometry and headspace^45^. For this reason, here we have investigated the effects of a 100 fold scale up, from 10 mL to 1 L, on the ionoSolv process as an intermediate step between bench- and pilot-scales. This is an efficient way of obtaining meaningful information of the behavior of the process at a scale that is still easy to handle at the laboratory scale but large enough to reveal potential drawbacks of the process when performed at larger scales.

Here, we address three key remaining challenges to reduce costs of IL pretreatment by process intensification: (1) increasing biomass loading, to minimize the consumption of chemicals and reactor size; (2) increasing biomass particle size, reducing energy required to grind biomass; and (3) process scale-up, investigating pretreatment efficacy after introduction of stirring and the adaptation of pulp and lignin washing and recovery protocols to handle larger process volumes.

## Experimental

### Study design

In this study, *Miscanthus* × *giganteus* was chosen as feedstock because it has been extensively studied in our laboratories and is considered suitable for large-scale biorefining, being geographically widespread and having low water and land requirements for cultivation^4,5,46^. We initially focus on process intensification of *Miscanthus* pretreatment at the bench scale (10 mL) by increasing the biomass loading from 2 to 50 wt% and the particle size from fine powder (smallest dimension < 1 mm), to medium fibres (< 2 mm) to large chips (< 10 mm). Adaptation of pulp washing protocols was explored to reduce lignin deposition onto the pulp surface. Finally, we investigate 100-fold scale-up in a 1-L stirred reactor while varying stirring speeds to improve heat and mass transfer. At this scale it was not possible to investigate loadings above 20 wt% due to limited stirrer motor power compatible with the acid-resistant glass reactors used. The outcome of all pretreatment experiments was assessed by gravimetric determination of the pulp and lignin yield, characterization of pulps by enzymatic saccharification and compositional analysis, and lignin analysis by HSQC NMR and GPC.

## Materials and method

*Miscanthus* × *giganteus* was obtained from Imperial College London Silwood Park campus (Berkshire, UK) in December 2014 and April 2016. Each set of experiments were conducted with biomass prepared during a single harvesting batch (batches were not mixed). All biomass was air-dried and either manually chopped or comminuted using a hammer mill (Retch SM 2000) followed by sieving (Retsch AS 200) to the relevant size fraction. Unless otherwise indicated, biomass was size-reduced using the hammer mill and sieved to 0.18–0.85 µm (− 20/+ 80 US mesh scale) as the standard particle size fraction.

Complete experimental details can be found in the ESI.

### Ionic liquid synthesis (triethylammonium hydrogensulfate, [TEA][HSO_4_])

All pretreatment experiments in this study used a solution of 80 wt% [TEA][HSO_4_] with 20 wt% deionized water as pretreatment solvent. Experiments conducted at the 10 mL scale used [TEA][HSO_4_] synthesized in batches of ~ 150 g following a method previously described^47^, with the exception of benchmarking experiments carried out as a direct comparison of scale-up experiments. Larger quantities for all scale-up and benchmarking experiments were synthesized in a continuous stirred tank reactor (CSTR) designed and built for continuous production of ~ 1 kg/h protic ILs (further details in the ESI). The acidity of the IL solutions was measured with a pH meter (Mettler Toledo SevenEasy). The thermogravimetric analysis of dried fresh IL (containing < 0.5 wt% water as verified by Karl-Fischer titration) was performed on a TA Q500 (TA Instruments, USA) TGA analyzer.

### Standard operating procedure: 10 mL scale pretreatment

For “bench scale” experiments (10 g of IL solution), ionoSolv pretreatment was carried out using a published standard operating procedure^47^. Biomass to solvent ratios of between 1:2 and 1:50 g/g (2–50 wt% loading) were used and *Miscanthus* was pretreated for 6 h at 120 °C in sealed glass pressure tubes. After the pretreatment time had elapsed, the pulp was separated by centrifugation, washed (4 × 40 mL per g of biomass) and Soxhlet extracted with ethanol (24 h) and air dried. The ethanol was evaporated from the IL/ethanol mixture and lignin was precipitated by addition of deionized water (3 equivalents, defined as a ratio of water to IL solution of 3 g/g), isolated by centrifugation, washed two more times, dried in vacuo at 45 °C overnight and weighed to determine the lignin yield.

For the hornification experiments, two triplicates sets of pretreated *Miscanthus* samples were subjected to the same ethanol washing steps until the Soxhlet step. In one set of triplicates, the pulp was air-dried after the Soxhlet extraction step and in the other it was washed with water and stored wet to avoid drying-induced hornification.

### Alternative washing protocols

For DMSO washing experiments, the pulps were first washed with ethanol (4 × 40 mL) as in the standard procedure. After the fourth ethanol wash, they were incubated in 40 mL of DMSO overnight, centrifuged (3000 rpm, 50 min), the DMSO decanted and the pulps were then Soxhlet extracted with ethanol following the standard procedure.

For experiments where pulps were saccharified without air-drying to avoid hornification, after the Soxhlet extraction step the pulps were left in the thimble inside a Falcon tube filled with DI water for one hour, centrifuged (3000 rpm, 50 min), the supernatant decanted and the washing step was repeated once more in DI water. The pulps were weighed, their moisture content determined and saccharification run the following day.

### Particle size experiments

Three sets of experiments with different particle sizes, prepared as explained in the ESI, were conducted for two different feedstock-solvent combinations: *Miscanthus*-[TEA][HSO_4_] (120 °C, 6 h), and *Miscanthus*-dilute acid (3 wt% H_2_SO_4_, 120 °C, 1.5 h). The standard pretreatment protocols for bench scale ionoSolv and dilute acid pretreatment, respectively, were followed.

### Benchmarking experiments

Benchmarking experiments were carried out in 15 mL pressure tubes manufactured by Ace Glass Inc. without stirring, using identical pretreatment conditions as the scaled-up experiments below (see Table ESI-1).

### Standard operating procedure: 1 L scale pretreatment

All scale-up reactions were carried out using the experimental set up described previously^5^, employing an ‘Ecoclave’ pressure reactor system on a 1.5 L jacketed borosilicate glass pressure vessel (Type 2, rated to 6 barg, Büchi AG, Switzerland, Figure ESI-1). The reactor was loaded with either 100 g or 200 g of *Miscanthus,* on oven dried basis, for 10 or 20 wt% loadings, respectively and 1 kg of IL. The biomass-IL slurry was heated to 120 °C with stirring at 150 rpm (unless otherwise indicated) from an anchor impeller. After the reaction, the IL-pulp slurry was vacuum filtered and washed with ethanol (10 × 1 L). The recovered IL and ethanol washes were concentrated by rotary evaporation until all ethanol had been removed. After ethanol removal, lignin was precipitated and washed with deionized water. The IL-water mixture remaining was collected and concentrated to determine the IL recovery rate. The recovered ethanol and water were separately collected and re-used in subsequent washing steps to reduce solvent wastage. Full experimental details, including a sample heating curve can be found in the ESI (Figure ESI-2).

### Compositional analysis

The compositions of the untreated biomass, pretreated pulps and precipitated lignins were determined according to the NREL protocol ‘Determination of Structural Carbohydrates and Lignin in Biomass’^48^. For pulps that were obtained both in hornified (dry) and non-hornified (wet) state, only the dried hornified pulps were analyzed by compositional analysis. The composition of the non-hornified pulps was assumed to be identical. For pretreated pulps arising from larger particle size experiments (> 0.85 mm), the materials size was reduced to ≤ 0.85 mm before the analysis so they could be fully digested during the acid hydrolysis step.

### Saccharification assay

Saccharification assays were carried out based on NREL protocol ‘Enzymatic saccharification of lignocellulosic biomass’^49^. Experiments were run in triplicate using Cellic^®^ CTec2 enzyme blend obtained from Novozymes (Denmark), an analytical enzyme mixture containing cellulases, β-glucosidases, and hemicellulase, for degrading cellulose to fermentable sugars (20 µL per sample). The initial rate of hydrolysis was determined by sampling at intervals of 0.5, 1, 2, 4, 24, and 72 h and final glucose concentrations were determined after 7 days (Figure ESI-3). Glucose yields were reported as a percentage of the total glucan content found in untreated biomass.

### Particle size distribution measurements

For all the particle size experiments, the particle size distributions were determined using nested sieves. The sample particle size distribution was obtained by shaking for 20 min on a vibratory sieve shaker equipped with stacked sieves with decreasing pore sizes. The percentage weight of material retained by each sieve was measured and the log-normal distribution mass median diameter (*D*_*50*_) was calculated. Particle size distributions were plotted as weight percentages of samples passing through different sieve pore sizes (on a log scale) and the geometric mean diameters *D*_*50*_ were compared.

### Characterization of lignins

HSQC experiments were recorded on a Bruker 600 MHz spectrometer, using ca. 20 mg of lignin and DMSO-d_6_ as solvent, for lignins isolated from selected experiments. Semi-quantitative data were obtained using shortened experiments and by comparing the peak volume of each correlation with respect to volume integration of the G_2_ + G_2,cond_ signal, used as internal standard^4^. GPC measurements were performed using an Agilent 1260 Infinity instrument equipped with a Viscotek column set (AGuard, A6000M and A3000M).

### Elemental analysis

Representative samples were selected from experiments conducted at 10 wt% and 50 wt% loadings. CHNS analysis of untreated air-dried biomass, pulp, lignin and recovered IL were performed in duplicate by MEDAC Ltd (Chobham, UK). Oxygen content was obtained by difference. Accuracy is ± 0.30% absolute. CHNSO compositions and the calculated elemental composition of fresh IL were presented on a dry basis and used to conduct an elemental mass balance on the systems.

## Results and discussion

The effect of increasing solids loading and particle sizes for pretreatment of *Miscanthus* × *giganteus* using the low-cost PIL [TEA][HSO_4_] was first examined at the bench scale in unstirred glass tubes (working volume of 10 mL). The pulp recovery protocol was modified to improve saccharification yields at high solids loadings (see the “benchmarking experiments” section on the SI). These results constituted baseline data for comparison with scale-up runs. Then, process intensification was investigated by 100-fold scale-up (working volume of 1 L). The efficacy of pretreatment at both scales was compared by analyzing the following key parameters: pulp and lignin yields, pulp composition, saccharification yields and kinetics, and lignin characteristics.

### Effect of biomass loading

We assessed the pretreatment efficacy at loadings ranging from 2 to 50 wt%, corresponding to solid to liquid ratios of 1:50 to 1:2 g/g. These experiments were carried out under non-optimal conditions (120 °C, 6 h) from the point of view of obtaining maximal glucose release in saccharification, where the differences in pretreatment efficacy would be easier to observe.

#### Pulp, lignin and saccharification yields

**Figure 1:**
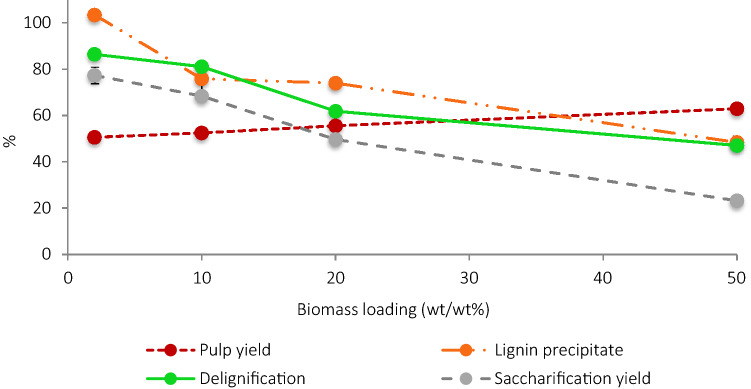
Comparison of the key pretreatment parameters—pulp yield, lignin and saccharification yields and delignification- after pretreatment of *Miscanthus* with [TEA][HSO_4_] (water content of 20 wt%) for 6 h at 120 °C at different biomass loadings, ranging from 2 to 50 wt%. Errors were calculated as standard deviation across triplicates.

Increasing the biomass loading from 2 to 50 wt% showed a steady increase in pulp yields from 51 to 63%, while lignin yields decreased from quantitative recovery at 2 wt% loading to 48.3% at 50 wt% loading (Fig. 1). Similarly, delignification and saccharification yield decreased gradually from 86 and 77%, respectively, to 47% and 23% in the same range. Lower saccharification yields could be due to a higher amount of lignin present in the pulp, as it has been previously suggested^50,51^. Some protic ILs can dissolve up to 70 wt% lignin^52^, making saturation unlikely, but the addition of ethanol can precipitate large fragments of lignin during the pulp wash^53^. Therefore, the decrease in lignin yields and delignification combined with the increase in pulp yields may be due to precipitation of lignin during the ethanol washing step, an idea reinforced by the results of the experiments with DMSO washing (see below). At higher loadings, the higher concentrations of dissolved lignin resulted in a greater amount of re-precipitation upon anti-solvent addition. To elucidate if this influences the initial hydrolysis rate, as redeposited lignin hinders the accessible surface area of the pulp for the enzymes, the kinetics of the enzymatic hydrolysis over a 7-day period of the pulps was investigated. Initial hydrolysis rate (glucose released in the first 2 h) and biomass digestibility after 7 days were estimated (Figure ESI-3, Table 1).

**Table 1:** Initial glucose release after 2 h and final glucose release after 7 days of hydrolysis.

	Initial hydrolysis rate (% h^−1^)^a^	7 day glucose yield (wt% glucan in untreated biomass)^b^	7 day glucose yield (wt% glucan in biomass pulp)^c^
Untreated *Miscanthus*	0.7 ± 0.1	12.2 ± 0.8	12.2 ± 0.8
2% loading	6.1 ± 0.7	77.3 ± 3.6	93.3 ± 5.1
5% loading	5.3 ± 0.1	73.3 ± 3.8	–
10% loading	5.2 ± 0.3	68.3 ± 1.1	78.4 ± 4.3
20% loading	3.7 ± 0.2	49.6 ± 1.5	60.1 ± 1.8
30% loading	2.8 ± 0.2	45.4 ± 1.7	–
40% loading	2.3 ± 0.4	34.5 ± 1.7	–
50% loading	1.5 ± 0.3	23.1 ± 0.5	26.5 ± 0.6

^a^Initial hydrolysis rate determined as glucose released after first 2 h of hydrolysis.

^b^^,^^c^Final glucose release after 7 day is expressed as two different percentages of glucose release: (b) relative to untreated biomass, and (c) relative to pretreated pulp.

The initial hydrolysis rate decreased from 6.1 to 1.5% glucose · h^−1^ when the loading was increased from 2 to 50 wt%. This may be related to the enzyme-accessible surface area of biomass, indicating a greater amount of re-precipitated lignin on the pulp surface hindering enzyme access to cellulose^54–56^. Final glucose yields after 7 days of enzymatic hydrolysis also decreased with biomass loading, attributed to higher residual lignin content of the pulp^18^. If the glucan yield is normalized to the glucan present in the pulp, rather than glucan in untreated biomass, at 2 wt% loading the pulp glucan was nearly quantitatively hydrolyzed but at 50 wt% loading only 27% was, which could again be attributed to lignin re-precipitation (Table 1). Similar results were reported with [Emim][OAc] pretreatments, which found decreased saccharification kinetics and final biomass digestibility at 50 wt% loading compared to 10 wt%^12^. However, for cellulose-dissolving ILs the possibility of lignin re-precipitation onto the pulp surface is limited and the negative effect of loading on saccharification was lower.

#### Lignin analysis

The molecular weights (M_W_) of the precipitated lignins were analyzed by GPC, while changes in interunit linkage and subunit abundance were analyzed by HSQC NMR. HSQC NMR of the recovered lignins suggest a slight trend towards greater ether cleavage with increasing solids loading (Figure ESI-7a). At higher loadings, signal intensities of the S, G_2_ and G_6_ positions appear to have decreased, while that of S_cond_ and G_cond_ increased slightly, indicating condensation reactions in G and S units. These differences, however, are not very pronounced. GPC data of the isolated lignins showed a general and gradual decrease in M_n_, M_w_ and polydispersity index (Ð) with higher loadings (Figure ESI-7b). Similar findings were reported with the PIL [HBim][HSO_4_] on the softwood pine^57^. This could be due to the method used to recover lignin, rather than the effect of higher loading. As previously stated, the addition of ethanol can precipitate larger lignin fragments during the pulp wash. This would become more pronounced at higher concentrations, where the cut-off for the precipitation of larger fragments would lie at lower M_W_^53^. It would lead to an apparent trend for increased ether cleavage without more pronounced condensation at higher loadings, while increasing pulp yield and decreasing lignin recovery. These results suggest that the lignin structure is not significantly altered by the use of high solids loadings. However, they show the need for an adapted washing protocol to either avoid re-precipitation or remove re-precipitated lignin from the pulp surface.

#### IL recovery

Thermal stability is a key property for IL recycling. [TEA][HSO_4_] was found to be thermally stable within the detection limits of ^1^H and ^13^C NMR at 150 °C for 100 h (Figure ESI-11). Hence, IL recyclability is expected to be dependent on IL recovery methods rather than its thermal degradation. The IL liquors produced after pretreatment at various biomass loadings were recovered after lignin precipitation, dried and weighed. IL recoveries approached 99 wt% for solid loadings of 10 and 20 wt% but decreased down to 92% with increasing biomass loadings up to 50 wt% (Figure ESI-12). A possible explanation could be due to a larger quantity of IL being trapped or bound within the lignin matrix, as shown by Brandt et al^44^ If this was the case, it should be detected in the N and S contents of the lignins. However, the elemental balance (Table ESI-4) shows that N and S content and total mass in the lignin was actually *lower* at higher loading, with residual IL contents of 4.2 and 3.9 wt% at loadings of 10 and 50 wt%, respectively, and did not account for the difference in IL recovery observed. Hence, the lower IL recovery was attributed to the lower total solvent volume used, so minor spillages or other losses accounted for a larger proportion of total mass. Solvent volumes were halved (from 10 to 5 mL) for experiments at high loadings in order to fit large quantities of biomass into 15 mL tubes. IL recoveries should improve upon process scale-up, where losses from IL sticking to glassware would account for a lower proportion of total IL.

Additionally, the pH values of a 1 wt% solution of fresh IL and IL liquors after use at various loadings were recorded and compared (Figure ESI-13) and the proton concentration in undiluted IL solutions was estimated (Table ESI-3). [H^+^] for the fresh IL was 0.22 mmol/g, while for recovered ILs [H^+^] ranged between 0.18–0.20 mmol/g, decreasing with increasing biomass loadings. After pretreatment at 10 and 50 wt% loading, 11 and 26 mol% protons were lost compared to the fresh IL solution, respectively. In line with previous findings that observed a 9 mol% decrease in proton concentration after four uses of the IL at 10 wt% loading^4^. This suggests that the acidity of the IL solution may need to be adjusted during repeated use, and that the “IL make-up stream” will predominantly consist of sulfuric acid, the far less expensive IL component^4^.

#### Mass balance

**Figure 2:**
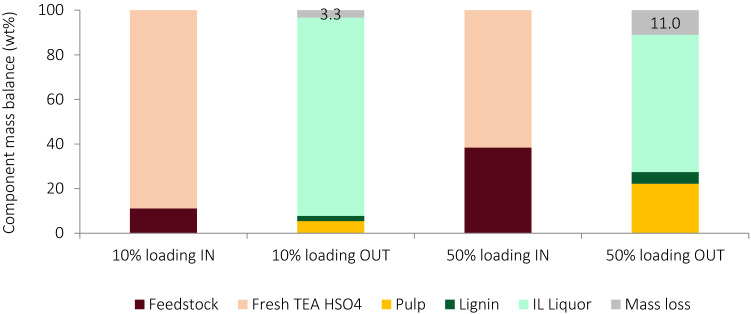
Total component mass balance for experiments conducted at 10 wt% and 50 wt% biomass loading. Results are shown per 100 g of starting material on a dry basis, assuming no IL losses.

The aforementioned observation of decreasing IL recovery prompted the analysis of the mass balance of the process. It was determined using the elemental compositions of the inputs (*Miscanthus* and fresh IL) and outputs (pretreated pulps, lignin and recovered IL). The total component mass balances for two experiments, conducted at 10 wt% and 50 wt% biomass loadings, are displayed in Fig. 2 and the elemental mass balances are shown in Table ESI-4.

The mass recovery decreased from 10 wt% loading (96 wt% recovery, 4 wt% loss) to 50 wt% loading (84 wt% recovery, 16 wt% loss). If IL recoveries are set to 100% (Table ESI-4), total mass losses account for 3.3 wt% and 11.0 wt% at low and high loadings, respectively. This is attributed to evaporation of volatile hemicellulose degradation products (acetic acid, formic acid, furfural and 5-HMF) from the liquor during concentration of the ethanol-IL washes and the removal of the water added during precipitation. It matches well with previous studies that found ~ 3.8 wt% lost in this form during processing at 10 wt% loading^4,57^. Even though a higher system mass was lost at higher loadings, the proportion of mass loss was slightly lower at higher than at lower loadings (287 mg/g vs 297 mg/g), suggesting the mass balance improved at higher biomass loadings.

#### Effect of pulp washing

**Figure 3:**
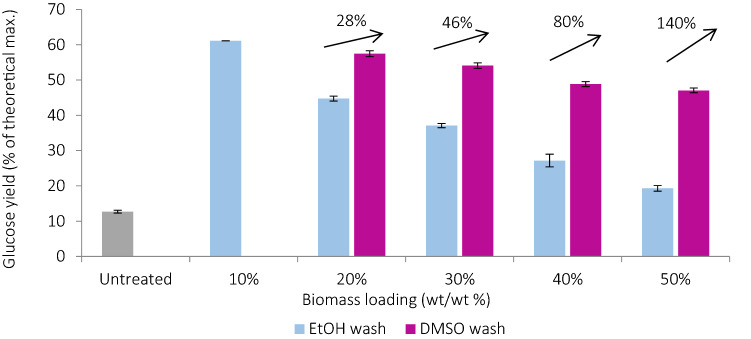
Glucose yields obtained after four ethanol washes (ethanol wash) and four ethanol washes plus one DMSO wash (DMSO wash) of air-dried pretreated *Miscanthus* pulps at various biomass loadings. Enzymatic hydrolysis was carried out for 7 days and glucose yields are shown relative to the amount of glucan in untreated biomass. Percentage increase following DMSO wash relative to ethanol wash is shown inset.

To maintain satisfactory saccharification yields at high solids loadings, the pulps were washed with DMSO after the ethanol wash. DMSO was selected as it is capable of dissolving re-precipitated lignin from the pulps surfaces, inexpensive (~ $1/kg)^58^ and fairly non-toxic. After incubation in DMSO overnight the solvent turned brown, suggesting lignin dissolution, and the pulps became lighter in color (Figure ESI-14). This effect was more pronounced for pulps pretreated at higher loadings, with more re-precipitated lignin. Enzymatic digestibility of the pulps was significantly enhanced. 47–58% glucose yields were obtained, a relative increase of 28–140% compared with ethanol-washed pulps (Fig. 3). DMSO washing was more effective for pulps produced at higher loadings. However, glucose yields still decreased with increasing loading, suggesting either that re-precipitated lignin may not be the only factor limiting pretreatment efficacy at higher loadings or that DMSO washing alone was insufficient to remove all lignin from the pulp surface. On the other hand, DMSO is a non-volatile solvent which would be highly energy-intensive to separate from the IL, which is undesirable for industrial processes as it can increase the operational costs. Other solvents are currently being investigated as alternative washing solvents and hot IL steam has been proposed as a future alternative for industrial-scale processes^51,56,59^.

#### Effect of hornification

Hornification refers to the physical change that happens in cellulosic materials after drying and that has been proposed to be caused by lactone bridge formation^60^. Pulp hornification upon air-drying can reduce enzymatic digestibility^61^. In industrial processes pulps would be enzymatically hydrolyzed in a wet state, so avoiding hornification is preferable to adding a DMSO-washing step. Therefore, pulps pretreated at 50 wt% loading were saccharified on a wet basis, which increased the glucose yield by 70%, releasing 66 wt% of glucose (Figure ESI-17). Yields were further improved to 74% with addition of a DMSO washing step before saccharification of the wet pulp.

### Effect of particle size

Comminution is highly energy-intensive and costly. Pretreating larger particles would reduce energy costs during biomass processing^25^. The particle size has a direct effect on the contact and diffusion of chemicals into the complex interior of the lignocellulose structure^34^. To evaluate the effect of particle size on pretreatment, three different particle size fractions (coarse, medium and fine) of *Miscanthus* × *giganteus* were investigated at 20 wt% loading (Table 2). Pretreatment with dilute acid (3 wt% H_2_SO_4_, 120 °C, 1.5 h) was also compared^62^.

**Table 2:** Particle size fractions for pretreatment of *Miscanthus* × *giganteus* after comminution by hammer milling^63–65^.

Particle size fraction	Description (*Miscanthus*)
Coarse	Cylindrical chips with dimension ~ 3 × 1 × 1 cm
	Size reduction by manual chopping
Medium	Fibers with dimension ~ 3 × 0.02 × 0.01 cm
	Size reduction by manual chopping
Fine	0.18–0.85 mm (standard size for previous experiments)
	Size reduction by cutting mill (2 mm sieve opening size) and sieving

**Figure 4:**
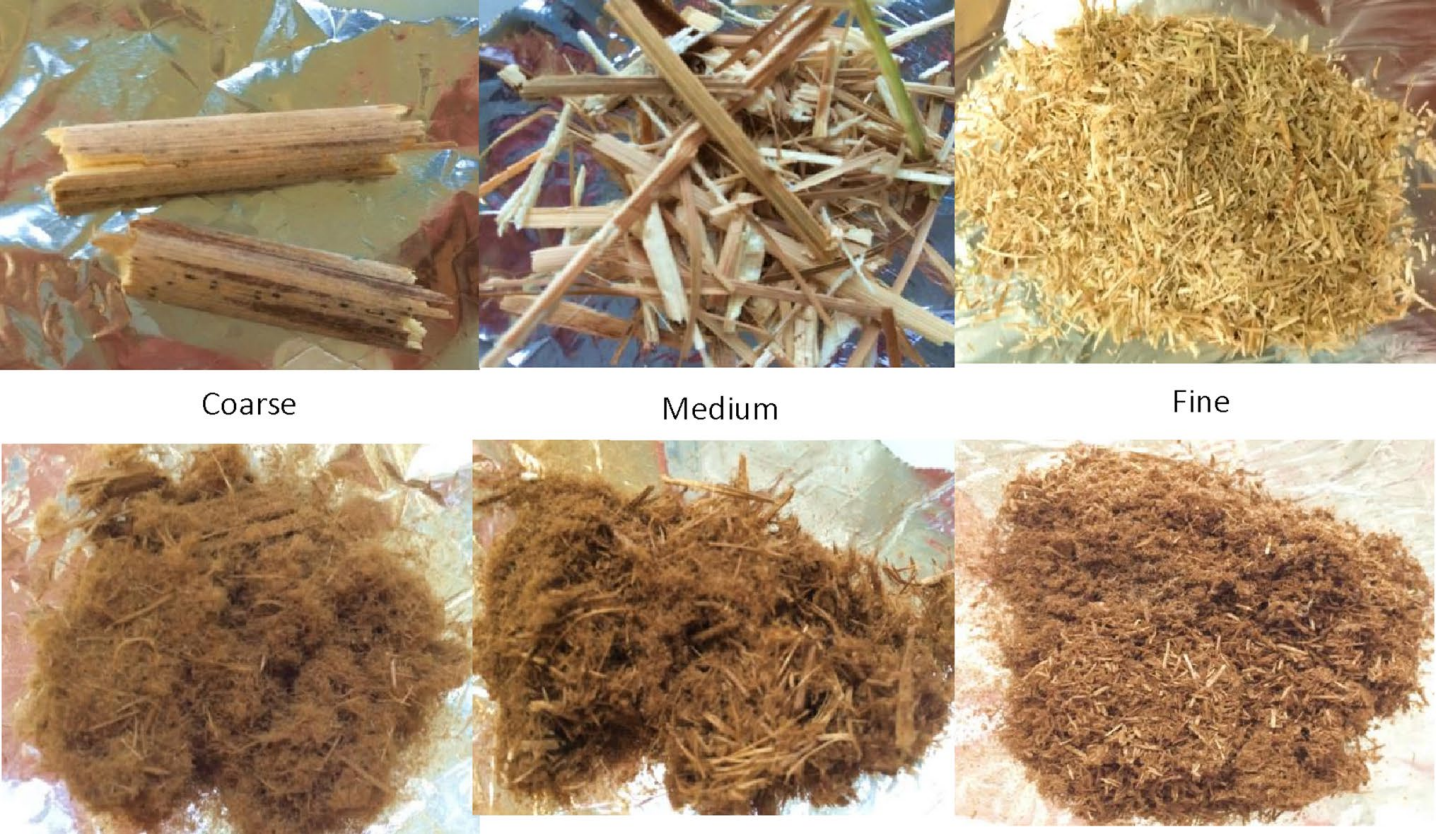
Photographs of (above) untreated biomass and (below) recovered pulps after pretreatment of *Miscanthus* with [TEA][HSO_4_] at 120 °C for 6 h at bench scale using a biomass loading of 20 wt%.

Chemical pretreatments, including ionoSolv, soften biomass structure by partially removing and modifying lignin and hemicellulose and reducing the particle size^23,66,67^. They are able to reduce size down to a certain “boundary”, which may be linked to the diameter of cellulose microfibril bundles released during hemicellulose and lignin solubilization^67,68^. Fiberization is reflected in the pulp images for *Miscanthus,* which had similar features regardless of initial particle size (Fig. 4).

The efficacy of pretreating different particle size fractions was evaluated by comparing the pulp and lignin recoveries, pulp delignification, glucose yields released by saccharification, and particle size distribution (PSD) of the pulps. While the enzyme-accessible surface area of the pulp is of most interest for explaining saccharification yields, its measurement is far more complicated than sieving, which was used as the preferred method to assess PSD changes in this study^36^. *Miscanthus* pulps were size-reduced during pretreatment (30–68%, based on the *D*_*50*_ values), due to its low recalcitrance and density (Figure ESI-18)^67,69^. Greater size reduction was observed for larger particles, though the change in *D*_*50*_ for the largest size fraction could not be quantified (Table 3). The medium and fine fractions were also pretreated using dilute sulfuric acid (3 wt%, 120 °C, 1.5 h). In this case, the recovered pulps closely resembled the starting materials and *D*_*50*_ values showed low size reduction for medium particles and virtually none for fine particles (Table 3, Figure ESI-19). On the other hand, ionoSolv pretreatment resulted in a dramatic degree of particle size reduction, with a more visible effect for the coarse chips (Fig. 4). This suggests that for low density grassy feedstocks, it may be possible to use pretreatment for initial size reduction followed by post-pretreatment size reduction to produce the fine particles needed for efficient hydrolysis and fermentation^36^, with added energy reduction due to the presence of the ionic liquid^70^.

**Table 3:** *D*_*50*_ values (in mm) obtained for untreated biomass and recovered pulps after IL and DA pretreatments.

Geometric mean average diameter *D*_*50*_	Coarse pulp	Medium pulp	Fine pulp
Miscanthus untreated	–	1.9 ± 0.1	0.37 ± 0.02
Miscanthus ionoSolv	1.0 ± 0.2 (–)	0.61 ± 0.01 (− 68%)	0.26 ± 0.01 (− 30%)
Miscanthus DA	–	1.7 ± 0.0 (− 11%)	0.4 ± 0.02 (~ 0%)

Particle size distributions were obtained by sieving. Values in brackets denote the percentage change in *D*_*50*_ values of the treated pulp based on the original untreated feedstock.

**Figure 5:**
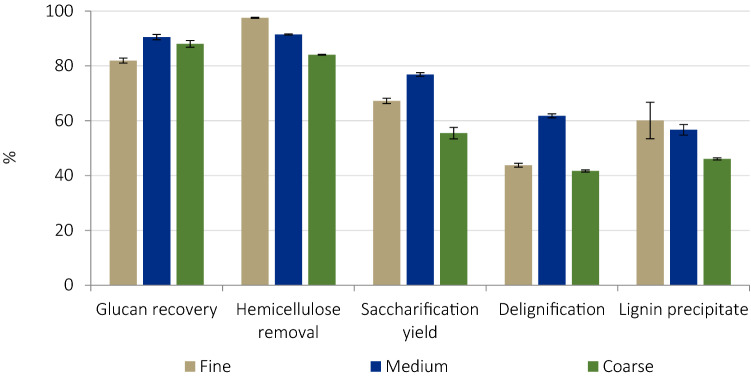
Effect of particle size of *Miscanthus* on key indicators for ionoSolv pretreatment, after pretreatment with [TEA][HSO_4_] at 120 °C for 6 h at bench scale using a biomass loading of 20 wt%.

Glucose yields were determined after grinding and sieving all pretreated pulps to 0.18–0.85 mm. No significant difference was seen in the glucan recovery values for coarse and medium particles (all ~ 90%), while a slightly greater degree of glucan loss was noted for fine particles (Fig. 5). This, previously observed for rice straw pretreatment^34^, suggests excess acidity in the IL^2^, which would have a greater effect for fine particles, with higher volumetric surface area exposed to the H^+^ protons. Hemicellulose extraction was more effective for finer particles due to greater sugar accessibility. Delignification showed a different trend, being greatest (60%) for medium particles. Coarse particles were less delignified due to the lower surface area to volume ratio; while fine particles also showed lower delignification than expected, presumedly due to re-precipitation of lignin onto the large pulp surface area. The lignin recovery, which was highest for fine particles, provides further support to this hypothesis. As a result, saccharification yields were higher for medium (77%) than for fine particles (67%). This suggests that the presence of lignin has a stronger negative effect on glucose yields than the positive effect of hemicellulose removal, as was previously noted^57^. These saccharification results were obtained for size-reduced pulps, which adds extra energy penalty but may be unnecessary for scale-up studies.

Based on this, the optimum size for unstirred *Miscanthus* pretreatment appears to be medium sized particles (~ 3 × 0.02 × 0.01 cm), in line with the proposed particle size range of 2–6 mm recommended by Cadoche and López to minimize comminution energy^21^.

Dilute acid treatment removed the majority of hemicellulose and small amounts of lignin (Figure ESI-21), as reported in literature^71,72^. However, delignification (~ 10%) and glucose yields (~ 28%) were very low for all size fractions*.* IonoSolv pretreatment offers many advantages over dilute acid pretreatment such as greater reduction in particle size, higher volumetric surface areas of the pulps, lower lignin contents, and higher lignin recoveries.

### Effect of 100-fold scale-up

Here, we demonstrate the 100-fold scale-up of the ionoSolv deconstruction of *Miscanthus*, relative to the bench scale (1 L vs 0.01 L) under the same conditions (120 °C, 6 h) and with solid loadings between 10–20 wt% (> 20 wt% could not be attempted due to stirrer motor limitations), using different particle sizes and stirring speeds. Insights into the effect of scale-up were garnered by comparing pulp and lignin recoveries, pulp composition and saccharification and lignin characteristics.

#### Pulp washing protocol optimization

The volume of solvent employed for pulp washing at the bench scale becomes impractical at larger scales. Material handling and pulp washing at larger scales is a critical operation that requires improvement^37,59^. Three methods were investigated to adapt the protocol for ~ 100 g of pulp: muslin cloth straining, centrifugation and vacuum filtration. Each washing step used 1 mL of ethanol per g of IL. Multiple washing steps with smaller volumes each should lead to better IL removal from the pulp with lower solvent requirements, as the constant partition coefficient for IL between the wash solvent and pulp is multiplied geometrically over multiple cycles. To assess this, the IL content in the solid fraction was measured to track the efficiency of washing steps, and the pulp samples were subjected to saccharification to evaluate the degree of inhibition by residual IL (Figure ESI-15).

Straining using a muslin cloth rapidly reduced IL content, giving acceptable digestibility after only 4 washes. However, further washing could not reduce the pulp IL content to below 6%, which could be problematic for downstream processing. Centrifugation required up to 16 washing steps to reduce the IL content to 8%. Also, glucose yields increased slowly and the pulps appeared clumpy and compressed (Figure ESI-16), which may limit washing efficacy. Vacuum filtration was the most effective, producing fluffy pulp that gave high glucose yields after only 6 washes. A steady decrease in IL content to 4% after 8 washes and < 0.5% after 12 washes was noted. Vacuum filtration with 10 washing steps was found optimal, giving high glucose digestibility, saving ~ 25% ethanol compared to bench scale and eliminating the need for a Soxhlet extraction step.

#### Product recoveries and pretreatment effectiveness

**Figure 6:**
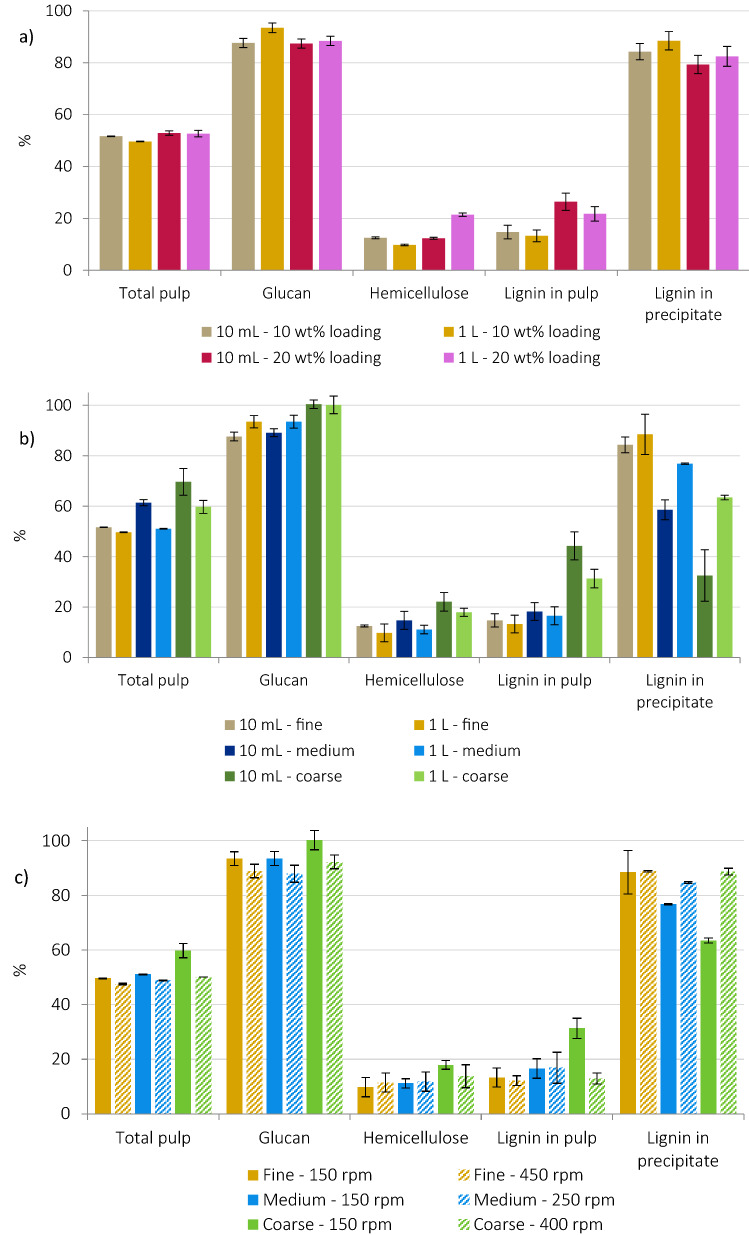
Comparisons of total solids (i.e. pulp), glucan, hemicellulose and lignin recovered in the pulp, and lignin precipitate yield (**a**) upon 100-fold scale-up for 10 and 20 wt% solid loading. Results shown as a proportion of component amount in untreated *Miscanthus*; (**b**) for fine, medium and coarse particle sizes. Results shown as a proportion of component amount in untreated *Miscanthus*; (**c**) for fine, medium and coarse particle sizes as a function of stirring speed. Results shown as a proportion of component amount in untreated *Miscanthus.*

Pulp yield recoveries for finely ground *Miscanthus* at 10 wt% and 20 wt% loadings (49.7% and 52.7%, respectively, Fig. 6a) were slightly lower at the 1 L scale than at the 10 mL scale (51.6% and 52.9%). Higher lignin precipitate yields, slightly higher delignification, hemicellulose removal, glucan recovery and improved glucose release were also seen at the 1 L scale. The exception being the hemicellulose extraction at 20 wt% loading (Fig. 6a, Table 4). These effects were attributed to improved heat and mass transfer upon the introduction of stirring.

**Table 4:** Key pretreatment outcomes at 10 mL vs 1 L scale as a function of particle size.

	Delignification (wt%)^a^	Lignin mass balance (wt%)^b^	Saccharification yield^c^ (%)	IL liquor recovery (wt%)
10 mL—fine—10% loading	85 ± 4	99 ± 6	79.9 ± 0.4	98.7 ± 0.6
1 L—fine—10% loading	87 ± 2	102 ± 6	79.1 ± 0.5	100.5 ± 0.6
10 mL—20% loading	74 ± 6	106 ± 7	60.8 ± 3.4	98.3 ± 0.4
1 L—fine—20% loading	78 ± 3	104 ± 7	63.2 ± 2.7	103.1 ± 0.3
10 mL—medium—10% loading	82 ± 4	77 ± 7	66.3 ± 6.5	101.6 ± 2.7
1 L—medium—10%	83 ± 4	93 ± 4	68.2 ± 4.8	100.9 ± 0.2
10 mL—coarse—10%	56 ± 6	77 ± 16	45.0 ± 3.6	99.9 ± 0.5
1 L—coarse—10%	69 ± 4	95 ± 5	59.3 ± 10.5	101.8 ± 0.6

^a^Based on feedstock lignin content (AIL + ASL), as determined by compositional analysis of untreated *Miscanthus.*

^b^Sum of residual lignin in pulp and isolated lignin yield.

^c^Glucose yield released from pulps as recovered after ethanol washing (without further size reduction).

At the 1 L scale, higher solids concentrations (20 wt% loading) increased the surface area for lignin re-precipitation onto the pulp, resulting in lower delignification, lignin and saccharification yields. However, this decrease in delignification and saccharification yield at 20% loading at the 1 L scale (9% and 16% lower, respectively) was less significant than at 10 mL scale (11% and 19% lower, respectively). Quantitative IL liquor recovery was also obtained.

The effect of different particle sizes was also compared for both scales. After scaling up more pronounced differences in product yields were found with increasing particle size (Fig. 6b, Table 4). The decrease in pulp yield upon up-scaling was more pronounced for medium and coarse fractions (around 10% drop at the 1 L compared to the 10 mL scale in both cases) than for fine particles (2% decrease). Lower pulp yields can be explained by improved lignin and hemicellulose extraction, as evidenced by compositional analysis. Stirred scale-up experiments also gave higher lignin precipitate yields, particularly for larger particle sizes.

All the pulps were subjected to saccharification without further size reduction (Table 5). Glucose yields for fine particles (80%) compared closely to those from 10 mL scale (79%). For medium particles a slight increase in glucose yield was found (68% vs 66%). Tellingly, the more drastic improvement was seen for coarse pulps (59% vs 45%), likely due to the improved mass transfer and size reduction during pretreatment with stirring. High uncertainty (~ 18% error) in the glucose yield for coarse particles was attributed to particle size heterogeneity. IL liquor recoveries were improved at the 1 L scale due to the larger solvent volumes used; recoveries exceeding 100 wt% are due to lignin fragments and other non-volatile extractives remaining in the liquor.

**Table 5:** Lignin characteristics at the 1 L scale as a function of solid loading and particle size.

	S/G ratio (–)	M_w_ (g/mol)	Ð (–)	β-*O*-4′ abundance
Fine—10%	0.70	4900	4.1	20.3
Fine—20%	0.72	5000	4.1	17.7
Medium—10%	0.69	5760	4.4	19.5
Coarse – 10%	0.79	6500	5.3	24.8

#### Insights from lignin HSQC NMR and GPC

The lignin precipitates obtained from stirred scale-up experiments for different loadings and particle sizes were analysed by HSQC NMR (Figures ESI-4, ESI-5 and ESI-6) and GPC. When increasing loading from 10 to 20 wt% at 1 L scale, precipitated lignins showed similar ether cleavage and condensation, as observed by levels of β-aryl ether, G_2_ and G_2,cond_ sub-units (Figure ESI-9). Different particle sizes, however, resulted in lignin with more distinct properties. For the largest particle sizes with lower volumetric surface area, IL diffusion into the particles and lignin diffusion out of the particles is slower, with lower proton/IL concentration at the particle core, reducing the rate of lignin extraction from the core and producing less depolymerized and less condensed lignins. This was seen from increasing β-*O*-4′ linkage abundance, increase in S and decrease in S_cond_ sub-units, and increase in S/G ratio as determined by HSQC NMR analysis of lignins extracted from coarse *Miscanthus* chips (Table 5, Figure ESI-9).

The precipitated lignins also had higher M_w_ for coarse (6500) than for fine particles (4900, Table 5). The increase in Ð values with particle size (from 4.1 to 5.3) could be explained by less depolymerized lignins being released from the core while smaller lignins still being released from the surface of the particles. Ð values were also higher than at the bench scale (2.7–3.0). These differences highlight the improvement in ionoSolv processing upon scale-up with stirring. Better mass transfer due to mixing may facilitate extraction and dispersion of lignin fragments in the IL.

#### Effect of stirring

The power requirements of mixing are non-negligible and demand optimization of slurry density and viscosity, mixing velocity and agitator design^7^. Different designs of agitator were tested and the most effective for mixing IL-biomass slurries, an anchor-shaped one, was selected. The effect of stirring speed on pretreatment was assessed by comparing stirring at 150 rpm and the maximum possible speed for a given slurry at 10 wt% loading (450 rpm for fine particles, 250 rpm for the medium particles and 400 rpm for coarse chips). Despite different ‘maximal’ stirring speeds, the maximum stirrer power was employed in all cases, therefore, the amount of energy delivered per second to the IL-biomass slurry was assumed to be the same.

Increasing stirring speed resulted in slightly lower pulp yield and higher lignin precipitate yield, suggesting improved lignin extraction (Fig. 6c). This became more prominent with increasing particle size. However, pulp compositions and saccharification yields were not significantly affected, with the exception of the saccharification yield of the fine fraction, which dropped drastically upon increasing stirring speed from 150 to 450 rpm (from 79.1 to 66.1%, Table 6).

**Table 6:** Key pretreatment outcomes at 10 mL vs 1 L scale as a function of stirring speed^a^.

	Delignification (wt%)	Lignin mass balance (wt%)	Saccharification yield (%)	IL liquor recovery (wt%)
Fine—150 rpm	87 ± 2	102 ± 6	79.1 ± 0.5	100.5 ± 0.6
Fine—450 rpm	88 ± 2	101 ± 2	66.1 ± 4.6	99.2 ± 0.6
Medium—150 rpm	83 ± 4	93 ± 4	68.2 ± 4.8	100.9 ± 0.2
Medium—250 rpm	83 ± 6	93 ± 6	67.8 ± 3.0	102.0 ± 0.2
Coarse—150 rpm	69 ± 4	95 ± 5	59.3 ± 10.5	101.8 ± 0.6
Coarse—400 rpm	87 ± 2	102 ± 3	59.0 ± 7.4	100.7 ± 0.6

^a^Selected stirring speeds: 150 rpm and maximal stirring speed that can be achieved for a particular experiment (250–450 rpm).

More rapid lignin extraction may take place with greater mixing due to improved heat and mass transfer. However, as fine particles have higher volumetric surface area, this increases the available pulp surface for lignin re-precipitation, worsened by particle size reduction during pretreatment. Consequently, overall delignification remained approximately constant with stirring speed (Table 6). However, for the coarse particle size fraction, increasing the stirring speed from 150 to 400 rpm improved delignification (from 69 to 87%) though glucose yields were very similar (59%). This indicates that mixing speed should be optimized depending on the particle size of the feedstock. The use of high stirring speeds at elevated temperatures for short residence times could help to maximize delignification and avoid prolonged contact of the pulp with the liquor to reduce lignin re-precipitation.

Faster mixing gave rise to greater in situ size reduction, producing pulps with lower average particle size and higher volumetric surface area (Figure ESI-20). *D*_*50*_ values showed a greater size reduction (40, 91 and 96% for fine, medium and coarse particles) than for unstirred experiments at the bench scale (30 and 68% for fine and medium particles).

The lignin precipitates recovered from 1 L scale experiments at different mixing speeds were analyzed by HSQC NMR and GPC. Only subtle differences in lignin characteristics were observed (Figure ESI-8). In all cases, faster mixing speeds produced lignins with lower abundance of β-*O*-4′ ether linkages, suggesting it is more cleaved. Also, a slight increase in signal intensity for G_6_ and G_2,cond_ subunits, and lower amounts of G_2_ and were seen, suggesting that lignins extracted using faster mixing were also more condensed. The implication, based on analysis of the lignin yields and structure, is that faster mixing led to a transition from “diffusion controlled” to “kinetically controlled” reaction regimes, leading to more condensation.

GPC analysis of the lignins (Table ESI-5) showed that increasing mixing speeds produced lignin precipitates with lower molecular weight, which may be due to a greater likelihood of lignin macromolecules precipitating onto the (increased) pulp surface area, though this is unclear. The different results in lignin yield, delignification and M_W_ depending on particle size illustrate the combined effects of lignin reactivity (producing more condensed lignins) and re-precipitation (reducing the proportion of high M_W_ lignins remaining in solution until the water addition step), both of which appear to become enhanced with stirring speeds.

## Conclusions

Process intensification of the ionoSolv process using the low-cost PIL [TEA][HSO_4_] and the grassy biomass *Miscanthus* was investigated, including the effects of biomass loading, particle size, 100-fold scaling up and stirring speed.

Five-fold increase in biomass loading (from 10 to 50 wt%) at the 10 mL scale decreased glucose yields from 68 to 23% due to re-precipitation of extracted lignin onto the pulp surface. The use of medium particle sizes (3 × 0.02 × 0.01 cm) was preferable over fine powders (0.18–0.85 mm), due to reduced surface area available for lignin re-precipitation, giving higher glucose yields while minimizing grinding energy needs. Comparison with dilute acid pretreatment showed ionoSolv processing is far more tolerant to use of larger particle sizes.

100-fold scale-up (from 10 mL to 1 L) improved the efficacy of ionoSolv pretreatment, after optimizing pulp washing protocols and agitator design. The introduction of stirring allowed higher delignification, reduced lignin condensation and improved lignin mass balance, particularly for larger particles (~ 3 × 1 × 1 cm). At this scale, doubling the solid loading from 10 to 20 wt% reduced lignin re-precipitation onto the pulp surface, giving higher glucose yields. IL liquor recovery rates were found to be > 99.2% in all cases. Substantial cost savings could be achieved by tailoring the pre-processing size reduction stages. Minimal stirring is preferable to minimize energy requirements.

Process intensification of stirred ionoSolv pretreatment has been demonstrated, showing great potential for further scaling up and optimization towards industrial application. Pulp washing protocols need further improvement to reduce both lignin precipitation and water requirements.

## Supplementary Information

Supplementary Information.

## Data Availability

All data generated or analysed during this study are included in this published article and its supplementary information files. Materials can be made available upon reasonable request to the author.

## Abbreviations

[DMBA][HSO_4_]: Butyldimethylammonium hydrogen sulfate
[Emim][OAc]: Ethylmethylimidazolium acetate
[TEA][HSO_4_]: Triethylammonium hydrogen sulfate
CAPEX: Capital expenditure
Comminution: Reduction of solid materials from one average particle size to a smaller average particle size
CSTR: Continuous stirred tank reactor
Ð: Polydispersity index
D_50_: Log-normal distribution mass median diameter
DMSO: Dimethyl sulfoxide
GPC: Gel permeation chromatography
HSQC: Heteronuclear single quantum coherence
IL(s): Ionic liquid(s)
NMR: Nuclear magnetic resonance
NREL: National Renewable Energy Laboratory
M_W_: Molecular weight
PIL(s): Protic ionic liquid(s)
PSD: Particle size distribution
rpm: Revolution per minute
TGA: Thermo gravimetric analysis
wt%: Weight percent

## References

[1] Humbird, D. *et al. Process Design and Economics for Conversion of Lignocellulosic Biomass to Ethanol*. *NREL Technical Report NREL/TP-5100-51400* vol. 303 http://www.nrel.gov/docs/fy11osti/51400.pdf%5Cnpapers2://publication/uuid/49A5007E-9A58-4E2B-AB4E-4A4428F6EA66 (2011).

[2] Verdía P, Brandt A, Hallett JP, Ray MJ, Welton T (2014). Fractionation of lignocellulosic biomass with the ionic liquid 1-butylimidazolium hydrogen sulfate. Green Chem..

[3] George A (2015). Design of low-cost ionic liquids for lignocellulosic biomass pretreatment. Green Chem..

[4] Brandt-Talbot A (2017). An economically viable ionic liquid for the fractionation of lignocellulosic biomass. Green Chem..

[5] Chambon CL (2020). Fractionation by sequential antisolvent precipitation of grass, softwood, and hardwood lignins isolated using low-cost ionic liquids and water. ACS Sustain. Chem. Eng..

[6] Baaqel H (2020). Protic ionic liquids—A case study in biomass. Green Chem..

[7] Klein-Marcuschamer D, Simmons BA, Blanch HW (2011). Techno-economic analysis of a lignocellulosic ethanol biorefinery with ionic liquid pre-treatment. Biofuels Bioprod. Biorefining.

[8] Zhang J, Hou W, Bao J (2015). Reactors for high solid loading pretreatment of lignocellulosic biomass. Adv. Biochem. Eng. Biotechnol..

[9] Zhang Y, Liu Y-Y, Xu J-L, Yuan Z-H, Zhuang X-S, He M-C (2012). High solid and low enzyme loading based saccharification of agricultural biomass. BioResources.

[10] Wood IP (2016). Comparison of saccharification and fermentation of steam exploded rice straw and rice husk. Biotechnol. Biofuels.

[11] Gschwend FJV (2019). Quantitative glucose release from softwood after pretreatment with low-cost ionic liquids. Green Chem..

[12] Cruz AG (2013). Impact of high biomass loading on ionic liquid pretreatment. Biotechnol. Biofuels.

[13] Viamajala S, Harvey OMS, Harvey SP (2010). Heat and mass transport in processing of lignocellulosic biomass for fuels and chemicals. Sustainable Biotechnology.

[14] Samaniuk, J. R. *Measurement and Modification of Biomass Rheological Properties, PhD Thesis, University of Wisconsin-Madison* (2012).

[15] Wu H (2011). Facile pretreatment of lignocellulosic biomass at high loadings in room temperature ionic liquids. Biotechnol. Bioeng..

[16] Selig MJ (2007). Deposition of lignin droplets produced during dilute acid pretreatment of maize stems retards enzymatic hydrolysis of cellulose. Biotechnol. Prog..

[17] Li H, Pu Y, Kumar R, Ragauskas AJ, Wyman CE (2014). Investigation of lignin deposition on cellulose during hydrothermal pretreatment, its effect on cellulose hydrolysis, and underlying mechanisms. Biotechnol. Bioeng..

[18] Laureano-Perez L, Teymouri F, Alizadeh H, Dale BE (2005). Understanding factors that limit enzymatic hydrolysis of biomass. Appl. Biochem. Biotechnol..

[19] Duff SJB, Murray WD (1996). Bioconversion of forest products industry waste cellulosics to fuel ethanol: A review. Biores. Technol..

[20] Sun Y, Cheng J (2002). Hydrolysis of lignocellulosic materials for ethanol production: A review. Bioresour. Technol..

[21] Cadoche L, López GD (1989). Assessment of size reduction as a preliminary step in the production of ethanol from lignocellulosic wastes. Biol. Wastes.

[22] Jacobson, J. J., Roni, M. S., Lamers, P. & Cafferty, K. G. *Biomass Feedstock Supply System Design and Analysis* (2014).

[23] Zhu JY, Pan XJ (2010). Woody biomass pretreatment for cellulosic ethanol production: Technology and energy consumption evaluation. Bioresour. Technol..

[24] Levey, S. Scientists save energy by lubricating wood. *Imperial College London News* http://www3.imperial.ac.uk/newsandeventspggrp/impe (2012).

[25] Hosseini SA, Shah N (2009). Multiscale modelling of hydrothermal biomass pretreatment for chip size optimization. Bioresour. Technol..

[26] Vidal BC, Dien BS, Ting KC, Singh V (2011). Influence of feedstock particle size on lignocellulose conversion—A review. Appl. Biochem. Biotechnol..

[27] Khullar, E. *Miscanthus conversion to ethanol: Effect of particle size and pretreatment conditions for hot water, PhD Thesis, University of Illinois at Urbana-Champaign* (2012).

[28] Bahcegul E (2012). Different ionic liquids favor different lignocellulosic biomass particle sizes during pretreatment to function efficiently. Green Chem..

[29] Li W (2011). Rapid dissolution of lignocellulosic biomass in ionic liquids using temperatures above the glass transition of lignin. Green Chem..

[30] Sun J (2016). Rapid room temperature solubilization and depolymerization of polymeric lignin at high loadings. Green Chem..

[31] Hou X-D, Li N, Zong M-H (2013). Facile and simple pretreatment of sugar cane bagasse without size reduction using renewable ionic liquids-water mixtures. ACS Sustain. Chem. Eng..

[32] Ren H, Zong M-H, Wu H, Li N (2016). Efficient pretreatment of wheat straw using novel renewable cholinium ionic liquids to improve enzymatic saccharification. Ind. Eng. Chem. Res..

[33] Papa G (2017). Parametric study for the optimization of ionic liquid pretreatment of corn stover. Bioresour. Technol..

[34] Nguyen T-AD (2010). Pretreatment of rice straw with ammonia and ionic liquid for lignocellulose conversion to fermentable sugars. Bioresour. Technol..

[35] Shi J (2013). Impact of mixed feedstocks and feedstock densification on ionic liquid pretreatment efficiency. Biofuels.

[36] Zhu W, Zhu JY, Gleisner R, Pan XJ (2010). On energy consumption for size-reduction and yields from subsequent enzymatic saccharification of pretreated lodgepole pine. Bioresour. Technol..

[37] Li C (2013). Scale-up and evaluation of high solid ionic liquid pretreatment and enzymatic hydrolysis of switchgrass. Biotechnol. Biofuels.

[38] Ferrari FA, Pereira JFB, Witkamp GJ, Forte MBS (2019). Which variables matter for process design and scale-up? A study of sugar cane straw pretreatment using low-cost and easily synthesizable ionic liquids. ACS Sustain. Chem. Eng..

[39] Yu C, Simmons BA, Singer SW, Thelen MP, VanderGheynst JS (2016). Ionic liquid-tolerant microorganisms and microbial communities for lignocellulose conversion to bioproducts. Appl. Microbiol. Biotechnol..

[40] Dickinson Q (2016). Mechanism of imidazolium ionic liquids toxicity in Saccharomyces cerevisiae and rational engineering of a tolerant, xylose-fermenting strain. Microb. Cell Fact..

[41] Li C (2015). Scale-up of ionic liquid-based fractionation of single and mixed feedstocks. BioEnergy Res..

[42] Weigand L (2017). Effect of pretreatment severity on the cellulose and lignin isolated from Salix using ionoSolv pretreatment. Faraday Discuss..

[43] Brandt, A. *et al.* An economically viable ionic liquid for the pretreatment of lignocellulosic biomass. *Green Chem.* 3078–3102 (2017) 10.1039/C7GC00705A.

[44] Brandt A, Chen L, van Dongen BE, Welton T, Hallett JP (2015). Structural changes in lignins isolated using an acidic ionic liquid water mixture. Green Chem..

[45] Malaret F, Gschwend FJV, Lopes JM, Tu WC, Hallett JP (2020). Eucalyptus red grandis pretreatment with protic ionic liquids: Effect of severity and influence of sub/super-critical CO_2_ atmosphere on pretreatment performance. RSC Adv..

[46] Gschwend FJV, Malaret F, Shinde S, Brandt-Talbot A, Hallett JP (2018). Rapid pretreatment of: Miscanthus using the low-cost ionic liquid triethylammonium hydrogen sulfate at elevated temperatures. Green Chem..

[47] Gschwend, F. J. V. *et al.* Pretreatment of lignocellulosic biomass with low-cost ionic liquids. *JoVE* e54246 (2016). 10.3791/54246. 10.3791/54246 PMC5091796 27583830

[48] Sluiter, A. *et al. Determination of Structural Carbohydrates and Lignin in Biomass*. *Biomass Analysis Technology Team Laboratory Analytical Procedure* vol. 2011 (2004).

[49] Resch, M. G., Baker, J. O. & Nrel, S. R. D. *Low Solids Enzymatic Saccharification of Lignocellulosic Biomass Low Solids Enzymatic Saccharification of Lignocellulosic Biomass Laboratory Analytical Procedure *(*LAP*). *Nrel* (2015).

[50] Berlin A (2006). Inhibition of cellulase, xylanase and beta-glucosidase activities by softwood lignin preparations. J. Biotechnol..

[51] Chen M (2020). Design of a combined ionosolv-organosolv biomass fractionation process for biofuel production and high value-added lignin valorisation. Green Chem..

[52] Rashid T, Kait CF, Regupathi I, Murugesan T (2016). Dissolution of kraft lignin using Protic Ionic Liquids and characterization. Ind. Crops Prod..

[53] Procentese A (2015). Deep eutectic solvent pretreatment and subsequent saccharification of corncob. Bioresour. Technol..

[54] Maurya DP, Singla A, Negi S (2015). An overview of key pretreatment processes for biological conversion of lignocellulosic biomass to bioethanol. 3 Biotech.

[55] Meng X, Ragauskas AJ (2014). Recent advances in understanding the role of cellulose accessibility in enzymatic hydrolysis of lignocellulosic substrates. Curr. Opin. Biotechnol..

[56] Nakasu PYS, Pin TC, Hallett JP, Rabelo SC, Costa AC (2021). In-depth process parameter investigation into a protic ionic liquid pretreatment for 2G ethanol production. Renew. Energy.

[57] Gshwend, F. J. V. *Towards an economical ionic liquid based biorefinery, PhD Thesis, Imperial College London* (2017).

[58] Alibaba. https://www.alibaba.com/showroom/dmso-price.html (last accessed 06/2021) (2020).

[59] Abouelela AR, Hallett JP (2021). Hazardous creosote wood valorization via fractionation and enzymatic saccharification coupled with simultaneous extraction of the embedded polycyclic aromatic hydrocarbons using protic ionic liquid media. ACS Sustain. Chem. Eng..

[60] Fernandes Diniz JMB, Gil MH, Castro JAAM (2004). Hornification—Its origin and interpretation in wood pulps. Wood Sci. Technol..

[61] Luo XL, Zhu JY, Gleisner R, Zhan HY (2011). Effects of drying-induced fiber hornification on enzymatic saccharification of lignocelluloses. Cellulose.

[62] Li C, Sun L, Simmons BA, Singh S (2013). Comparing the recalcitrance of eucalyptus, pine, and switchgrass using ionic liquid and dilute acid pretreatments. Bioenergy Res..

[63] Naimi LJ, Collard F, Bi X, Lim CJ, Sokhansanj S (2016). Development of size reduction equations for calculating power input for grinding pine wood chips using hammer mill. Biomass Convers. Biorefinery.

[64] Miao Z, Grift TE, Hansen AC, Ting KC (2011). Energy requirement for comminution of biomass in relation to particle physical properties. Ind. Crops Prod..

[65] Yancey NA, Tumuluru JS, Wright CT (2013). Drying, grinding and pelletization studies on raw and formulated biomass feedstock’s for bioenergy applications. J. Biobased Mater. Bioenergy.

[66] Brandt A, Gräsvik J, Hallett JP, Welton T (2013). Deconstruction of lignocellulosic biomass with ionic liquids. Green Chem..

[67] Tu WC (2020). Characterisation of cellulose pulps isolated from *Miscanthus* using a low-cost acidic ionic liquid. Cellulose.

[68] Stelte, W. *Steam explosion for biomass pre-treatment, Danish Technological Institute Report, Center for Renewable Energy and Transport*. *Danish Technological Institute Report, Center for Renewable Energy and Transport* (2013).

[69] Zhu JY (2011). Physical pretreatment—Woody biomass size reduction—For forest biorefinery. ACS Symp. Ser..

[70] Brandt A (2012). Soaking of pine wood chips with ionic liquids for reduced energy input during grinding. Green Chem..

[71] Pu Y, Hu F, Huang F, Davison BH, Ragauskas AJ (2013). Assessing the molecular structure basis for biomass recalcitrance during dilute acid and hydrothermal pretreatments. Biotechnol. Biofuels.

[72] Li C (2010). Comparison of dilute acid and ionic liquid pretreatment of switchgrass: Biomass recalcitrance, delignification and enzymatic saccharification. Bioresour. Technol..

