# Supplementary material for: Process intensification of the ionoSolv pretreatment: effects of biomass loading, particle size and scale-up from 10 mL to 1 L
Source: Sci Rep. 2021 Jul 28;11:15383. doi: 10.1038/s41598-021-94629-z (PMC8319198; doi:10.1038/s41598-021-94629-z)
Supplement: Supplementary file 1 — Supplementary Information. [file 41598_2021_94629_MOESM1_ESM.docx]

**Electronic supplementary information (ESI)**

**Process intensification of the Ionosolv Pretreatment: Effects of biomass loading, particle size and 100-fold scale-up**

Clementine L. Chambon^1^, Pedro Verdía^1^, Paul S. Fennell^1^, Jason P. Hallett^1,a^

^1^ Department of Chemical Engineering, Imperial College London, Exhibition Road, South Kensington, London, SW7 2AZ, United Kingdom

^a^ Correspondence: Prof. Jason P. Hallett, j.hallett@imperial.ac.uk, +44 2075943992

**Contents**

[Biomass comminution 2](#_Toc73995333)

[IL synthesis (scaled-up method) 2](#_Toc73995334)

[IL pretreatment at bench scale (10 mL) 2](#_Toc73995335)

[Scale-up of IL pretreatment (1 L) 3](#_Toc73995336)

[Compositional analysis 6](#_Toc73995337)

[Saccharification assay 7](#_Toc73995338)

[Characterization of lignins 8](#_Toc73995339)

[Thermal stability of [TEA][HSO_4_] 14](#_Toc73995340)

[IL recovery quantification 16](#_Toc73995341)

[Ionic liquid solution acidity 16](#_Toc73995342)

[Pulp washing optimization 17](#_Toc73995343)

[Effect of hornification 19](#_Toc73995344)

[Particle size distribution measurements 19](#_Toc73995345)

[Dilute acid pretreatment 22](#_Toc73995346)

[Mass balance 23](#_Toc73995347)

[Effect of stirring 25](#_Toc73995348)

[References 25](#_Toc73995349)

### Biomass comminution

The different size fractions of *Miscanthus* were prepared as follows: cylindrical chips of average size of 24 mm length × 9.9 mm diameter were cut by manual chopping using a band saw. Smaller fractions were prepared using a cutting mill (Retsch SM 2000) and a square sieve opening of 20 mm followed by sieving (Retsch AS 200) to 1–3 mm size fraction. The finest particles were prepared by grinding in the same equipment using a sieve opening of 0.75 mm followed by sieving to 0.18–0.85 µm (-20/+80 US mesh scale) size fraction. All biomass fractions were stored in plastic bags at room temperature in the dark.

### IL synthesis (scaled-up method)

Larger quantities of [TEA][HSO_4_] were synthesized in a continuous stirred tank reactor (CSTR) designed and built for continuous production of ~1 kg/h protic ILs. It was made of corrosion-resistant materials, namely a PTFE-lined jacketed vessel (4” nominal bore × 430 mm long, ASME 150, Corrosion Resistant Products Ltd, UK) cooled by a water jacket with PFA-lined Swagelok fittings. The reactor was fitted with stirring, temperature and conductivity controls to monitor the product quality. Dilute acid and amine were fed continuously into the reactor and reacted to form a solution with a final water content of 20 wt%. The CSTR was used to synthesize a batch of ~18 kg of [TEA][HSO_4_]_80%_ solution which was used for all scale-up and benchmarking experiments.

### IL pretreatment at bench scale (10 mL)

For “bench scale” experiments (10 mL working volume), ionoSolv pretreatment was carried out using a standard operating procedure.^1^ Biomass to solvent ratios of between 1:2 and 1:50 g/g were used (2–50 wt% loading). The experimental temperatures ranged between 120 and 170 °C with residence times between 0.5 and 6 hours.

For the biomass loading experiments, *Miscanthus* samples were pretreated with [TEA][HSO_4_]_80%_ at 120 °C for 6 h, using solid loadings of 2, 5, 10, 20, 30, 40 and 50 wt%. 2 wt% biomass loading was achieved by using 25 g of IL and 0.5 g of biomass; 5, 10 and 20 wt% loading were achieved using 10 g of IL and 0.5, 1 and 2 g of biomass respectively; 30, 40 and 50 wt% loading were achieved by using 5 g of IL and 1.5, 2 and 2.5 g of biomass, respectively.

#### Benchmarking experiments

Benchmarking experiments were carried out using IL ([TEA][HSO_4_]_80%_) from the same batch and identical pretreatment conditions (120 °C for 6 hours) as in scale-up experiments (Table ESI-1). After pretreatment, tubes were cooled and the pulps were washed by vacuum filtration (Whatman© grade 1, diam. 110 mm) using 1 mL/g IL solvent per wash over 12 washing steps. The pulp was air-dried and weighed before being subjected to saccharification *without* a Soxhlet step. The ethanol-IL washes were concentrated, and lignin was recovered as in the standard procedure.

### Scale-up of IL pretreatment (1 L)

#### Scale-up experimental setup

All scale-up reactions were carried using the experimental equipment and set up published recently,^2^ an ‘Ecoclave’ laboratory pressure reactor system supplied by Ken Kimble Ltd, UK (Figure ESI-1a). A 1.5-L jacketed glass pressure vessel with Hastelloy C22 internal components, a 50 mm anchor agitator and a high-torque overhead mechanical stirrer motor, the relief valve set to 1.7 barg and a bursting disc rated for 3 barg was used for all experiments.


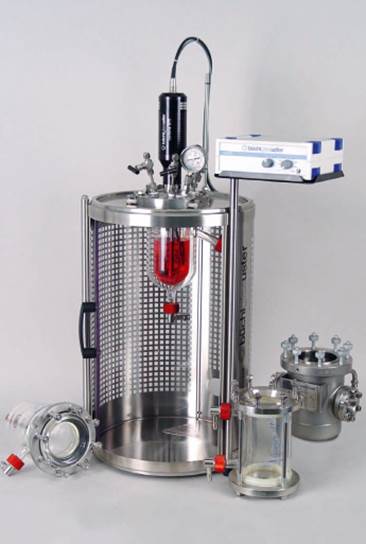


Coverplate with pressure fittings

Borosilicate glass pressure vessel

Additional pressure vessels

Magnetic drive fitted with motor (not shown)

Ecoclave reactor frame

Polycarbonate sliding door

**1 ×100**


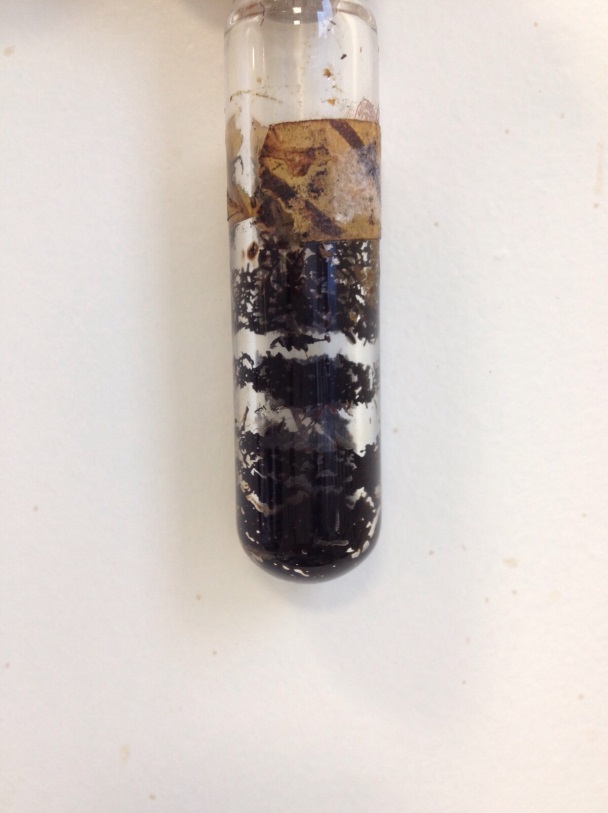


**10 mL**


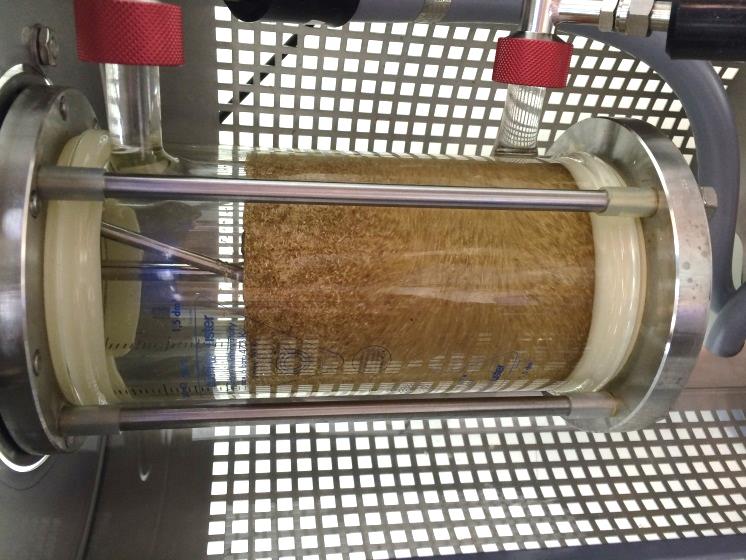

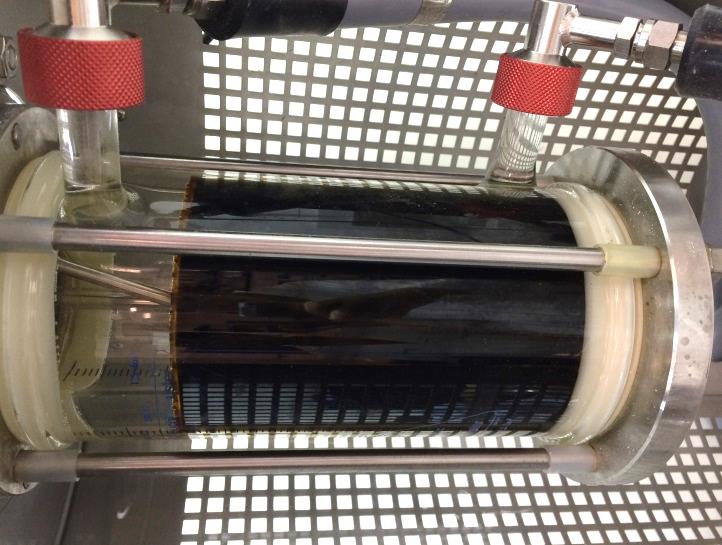


**1 L**

**1 L**


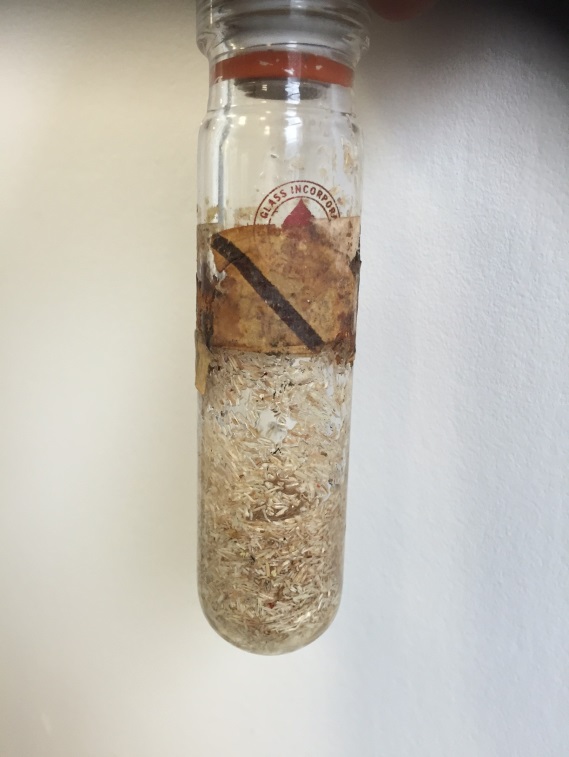


**10 mL**

Figure ESI-1. (a) Ecoclave reactor system used for 1 L-scale scale-up experiments showing key components; (b) IL-biomass slurry before/after pretreatment showing 15 mL vessel without stirring used at the 10 mL-scale (left) and 1.5-L glass pressure vessel with stirring used at the 1 L-scale (right). Images not to scale.

#### Standard operating procedure: 1 L-scale

IonoSolv pretreatment was performed in the 1.5-L glass vessel at constant agitation (150 rpm) and indirect heating via an oil jacket using the same basic operational procedure recently published.^2^ The glass reactor was loaded with either 100 g or 200 g of bone-dry *Miscanthus* (107 or 214 g of air-dried *Miscanthus*) for solid loadings of either 10 or 20 wt%, respectively. The biomass-IL slurry was then heated to the desired reaction temperature of 120 °C with a stirring speed of 150 rpm (unless otherwise indicated) from the anchor impeller; the heat-up time varied between 30 and 45 min, depending on the viscosity of the slurry. A sample heating curve is shown below (Figure ESI-2). The following experiments were conducted in triplicates: Fine/10wt% loading/450 rpm; Fine/20wt% loading/150 rpm; Medium/10 wt% loading/250 rpm; Coarse/10wt% loading/400 rpm; all other experiments (Fine, Medium and Coarse at 10 wt% loading and 150 rpm) were conducted in monoplicate. Errors were calculated as the standard deviation across triplicates. Percentage errors from triplicate experiments at the closest experimental conditions were used in the case of monoplicate experiments. After 6 h the reactor was vented, the oil jacket dismantled and the reactor allowed to cool down with the help of a fan. Cooling times to below 100 °C and below 60 °C were approximately 30 and 90 minutes, respectively. Detailed experimental conditions used for IL pretreatment are summarized in Table ESI-1.

Figure ESI‑2. Sample heating curve and process pressure for [TEA][HSO_4_]-biomass slurry (20 wt% loading) from 85 to at 150 °C using an oil jacket in a 1 L-glass reactor.

Table ESI‑1. Pretreatment parameters used for ionoSolv pretreatment scale-up studies and corresponding bench-scale experiments.

| **Factor** | **Low** | **Medium** | **High** |
| --- | --- | --- | --- |
| Scale (mL) | 10 | 1000 | - |
| Biomass loading (wt%) | 10 | 20 | - |
| Stirring speed | 150 | max ^a^ | - |
| Particle size | Fine (180 – 850 *μ*m) | Medium (1 – 3 mm) | Coarse (24 mm) |

^a^ The maximum stirring speed that could be achieved for each IL-biomass slurry, i.e. 450 rpm (fine/10 wt% loading), 150 rpm (fine/20 wt% loading), 250 rpm (medium/10 wt% loading) and 400 rpm (coarse/10 wt% loading).

After cooling of the reaction mixture, the pretreated IL-pulp slurry was manually transferred to a 5 L plastic container and washed with 1 L of absolute ethanol. The slurry was vacuum filtered (Whatman© grade 1, diam. 150 mm) and 10 washing steps were conducted in total, each step consisting of 1 L of EtOH (1 g EtOH/g IL solvent, since 1 L of IL-water mixture was used for each scale-up experiment). The recovered IL and ethanol washes were concentrated by rotary evaporation until all ethanol had been removed. The recovered ethanol was collected and re-used for washing in subsequent washing steps to reduce solvent wastage. Concentrated IL liquors showed signs of lignin precipitation if ethanol-rich washes were added to IL-rich liquors, as pure ethanol is a known precipitant for lignin.^3^ Therefore, the IL liquors were mixed after concentration. After all ethanol had been removed DI water was added to the concentrated IL liquor (i.e. 4 g/g of water to IL solvent mixture) to precipitate the lignin, which was left to equilibrate overnight.

Lignin recovery was carried out by two rounds of centrifugation in four 500 mL centrifuge tubes (Corning, USA) at 3000 rpm for 50 minutes to sediment the precipitated lignin. The lignin solid was then washed four more times, each with 2 L of DI water, until a total of ~10 L of water had been used for precipitation and washing. The supernatants (IL and water) were collected separately and concentrated to determine the IL recovery as given in Eq 1 (below). The recovered water was collected and recycled for lignin precipitation and washing in subsequent experiments to reduce solvent volumes. The recovered solid lignin was dried *in vacuo* at 45 °C for 48 h in the centrifuge tubes before being transferred to pieces of foil and re-dried for 24 h to fully remove any residual water, before determination of the lignin yield.

### Compositional analysis

Compositional analysis was carried out according to the NREL protocol ‘Determination of Structural Carbohydrates and Lignin in Biomass’.^4^ All biomass samples, untreated or pretreated, were Soxhlet extracted for 24 hours with ethanol and vacuum-dried for 48 h prior to compositional analysis.

Pulp compositional analysis was typically used to enable detection of cellulose, hemicelluloses and residual lignin, though the latter cannot be distinguished from reprecipitated lignin and pseudo-lignins. The pulp composition was used to calculate the glucan recovery, delignification and hemicellulose removal; these were used together with the lignin yield and sugar yield from enzymatic saccharification as the key indicators of pretreatment performance.

The delignification was calculated using Equation 3:

$$Delign.=\frac{{Lignin}_{untreated}-{(Lignin}_{pulp}\times{Yield}_{pulp})}{{Lignin}_{untreated}}$$

(Equation 3)

where *Lignin_untreated_* is the lignin content in untreated sugarcane bagasse, *Lignin_pulp_* is the lignin content in the pulp and *Yield_pulp_* is the oven-dried yield of pulp.

Glucan recovery in the recovered solids, i.e. the glucan detected during pulp analysis compared to glucan detected during analysis of native biomass, was calculated using Equation 4:

$$Glucan recovery=\frac{{Gluc}_{untreated}-{(Gluc}_{pulp}\times{Yield}_{pulp})}{{Gluc}_{untreated}}$$

(Equation 4)

whereby *Gluc_untreated_* is the glucan content of untreated sugarcane bagasse, *Gluc_pulp_* is the glucan content in the pulp and *Yield_pulp_* is the oven-dried yield of pulp. We note that glucan is a collective term given to D-glucose polymers made up of dehydrated glucose units; the majority of glucan in lignocellulosic biomass is in the form of cellulose.

Hemicellulose removal was calculated using Equation 5:

$$Hemicellulose removal=\frac{{Hem}_{untreated}-{(Hem}_{pulp}\times{Yield}_{pulp})}{{Hem}_{untreated}}$$

(Equation 5)

where-by *Hem_untreated_* is the hemicellulose sugar content in untreated sugarcane bagasse, *Hem_pulp_* is the hemicellulose content in the pulp and *Yield_pulp_* is the oven-dried yield of pulp.

For hornified and non-hornified pulps, only the dried hornified ones were analyzed by compositional analysis and the composition of the non-hornified pulps was assumed to be identical. For pretreated pulps arising from larger particle size experiments (>0.85 mm), the materials were size-reduced using an analytical grinder with stainless steel cutter (Yellow Line A10, IKA Works Inc.) prior to analysis. The samples were ground for up to 30 seconds as this was sufficient to grind the *Miscanthus* and pine chips to a powder that would pass through an 0.85 mm sieve opening. Compositional analysis of pretreated larger particle sizes was conducted after grinding of the pulps to ≤0.85 mm so that all pulps could be fully digested during the acid hydrolysis step of the analytical method. Delignification, glucan recovery and hemicellulose recovery were calculated as described above.

### Saccharification assay

Saccharification assays were carried out in triplicate according to the NREL protocol ‘Enzymatic saccharification of lignocellulosic biomass’,^5^ using Cellic® CTec2 enzyme blend obtained from Novozymes (Denmark). This analytical enzyme mixture contains cellulases, ß-glucosidases, and hemicellulase for degrading cellulose to fermentable sugars.

For wet samples, moisture contents were determined immediately before saccharification. Incubation was performed into a Stuart Orbital Incubator (S1500) at 50 °C and 250 rpm for 7 days, after which the glucose concentrations were determined. Time-point samples were also taken for certain samples at intervals of 0.5, 1, 2, 4, 24, and 72 hours to determine the initial rate of hydrolysis. Glucose yields were reported as a percentage of the total glucan content found in untreated biomass as determined by compositional analysis. Saccharification end point samples were obtained by filtering 1 mL of the saccharification mixture though a PTFE syringe filters. Samples were analyzed on a Shimadzu HPLC system with refractive index (RI) detector and an Aminex HPX-87P column (BioRad, 300 x 7.8 mm) with purified water as mobile phase (0.6 mL/min). The column temperature was 85 °C and acquisition time was 40 min. Calibration standards with concentrations of 0.1, 1, 2 and 4 mg/mL of each of glucose, xylose, mannose, arabinose and galactose, and 8 mg/mL of glucose were used. Glucose yields were reported as a percentage of the total glucan content found in untreated biomass (as determined by compositional analysis).

Glucose and xylose yields were calculated as a function of saccharification residence time *t* according to Equations 6 and 7, respectively:

$\%Glucose yield (t)= \frac{c_{HPLC,t}\times V\times0.9\times Y_{pulp}}{G_{0}\times{ODW}_{sample}}\times100$ (Equation 6)

$\%Xylose yield (t)= \frac{c_{HPLC,t}\times V\times0.88\times Y_{pulp}}{G_{0}\times{ODW}_{sample}}\times100$ (Equation 7)

where *c_HPLC_* is the sugar concentration detected by HPLC after saccharification time *t* had elapsed, *V* is the initial volume of the solution in mL (10.00 mL), *Y_pulp_* is the pulp yield recovered as a proportion of initial biomass dry weight before pretreatment (100% for untreated biomass; ~40–70% for pretreated pulps) *G_0_* is the glucan content (%) in untreated biomass, and *ODW_sample_* is the oven-dried weight of the biomass sample before saccharification in mg.

Figure ESI-3. Effect of biomass loading on enzymatic hydrolysis of pretreated Miscanthus × giganteus at 120 °C for 6.

### Characterization of lignins

#### HSQC NMR spectroscopy

For HSQC experiments of precipitated lignin, ca. 20 mg of lignin was dissolved in 0.25 mL of DMSO-d_6_ and the solution transferred to a Shigemi tube. HSQC NMRs were recorded on a Bruker 600 MHz spectrometer (pulse sequence hsqcetgpsi2, spectral width of 10 ppm in F2 (^1^H) with 2048 data points and 160 ppm in F1 (^13^C) with 256 data points, 16 scans and 1 s interscan delay).

HSQC experiments were recorded in monoplicate for *Miscanthus* lignins isolated from selected scale-up experiments.

Due to the difficulty in obtaining fully quantitative data, hindered by the long relaxation time of the ^13^C nucleus, semi-quantitative data were obtained using shortened experiments and by comparing the peak volume of a certain correlation with respect to another correlation that is considered constant over a range of experiments. Previous results reported by Gschwend et al. have shown that, despite the presence of other phenolic moieties, the G_2_ + G_2,cond_ integral is very stable over a 23 h time period for *Miscanthus* lignin and can be used as internal standard.^8^ Therefore, here the abundance of different sub-units and linkages in *Miscanthus* lignin are reported by using volume integration with reference to the G_2_ + G_2,cond_ integral. Sub-structural information was obtained by assigning the various signals and estimating the S_2,6_/G_2_+G_2cond_ ratio according to previous assignments.^9–11^ Here we are highlighting both the the aromatic (δC/δH: 110–130/6.0–9.0 ppm) and side chain regions (δC/δH: 50–90/2.5–5.8 ppm). The most common unsaturated subunit structures, including syringyl (S2,6,Scond.) and guaiacyl (G2,G5,G6,Gcond.) units can be analyzed from the aromatic region peaks, while the three most common linkages, β-O-4 ether (A), β–β(resinol, B) and β-5 (phenylcoumaran, C) can be analyzed on the side chain region (see table ESI-2 for the assignment of these signals).

Full spectra are presented below.

Table ESI‑2. Assignment of the most important ^13^C-^1^H cross-signals in the HSQC NMR spectra.

| **Label** | **δC /δH (ppm)** | **Assignment** |
| --- | --- | --- |
| β-O-4' | 70.93/4.83 | C_α_-H_α_ in β-O-4 substructures (A) |
| β-β' | 84.71/4.62 | C_α_-H_α_ in β-β (resinol) substructures (B) |
| β-5' | 87.08/5.51 | C_α_-H_α_ in phenyl coumaran substructures (C) |
| S_2,6_ | 102.95/6.75 | C_2_-H_2_ and C_6_−H_6_ in syringyl units (S) |
| S_cond_ | 106.01/6.33 | C_6_−H_6_ in condensed syringyl units (S) |
| G_2_ | 110.47/6.95 | C_2_-H_2_ in guaiacyl units (G) |
| G_2cond_ | 112.17/6.67 | C_2_-H_2_ in condensed guaiacyl units (G) |
| G_5_ | 115.23/6.56 | C_5_-H_5_ in Guaiacyl units (G) |
| G_6_ | 119.85/6.7 | C_6_-H_6_ in guaiacyl units (G) |


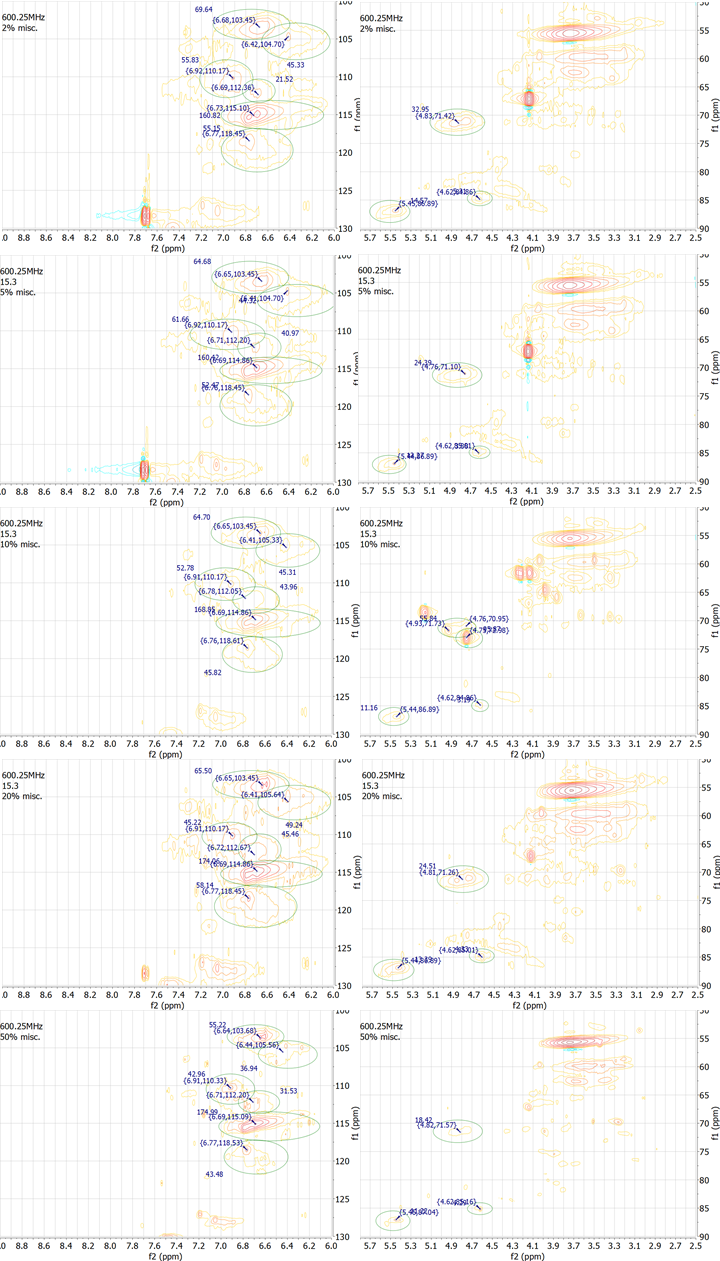


Figure ESI-4. HSQC spectra of the aromatic (left) and side chain (right) regions of IonoSolv lignins of Miscanthus at different solid loadings. From top to botton: 2 wt%, 5 wt%, 10 wt%, 20 wt% and 50 wt%.


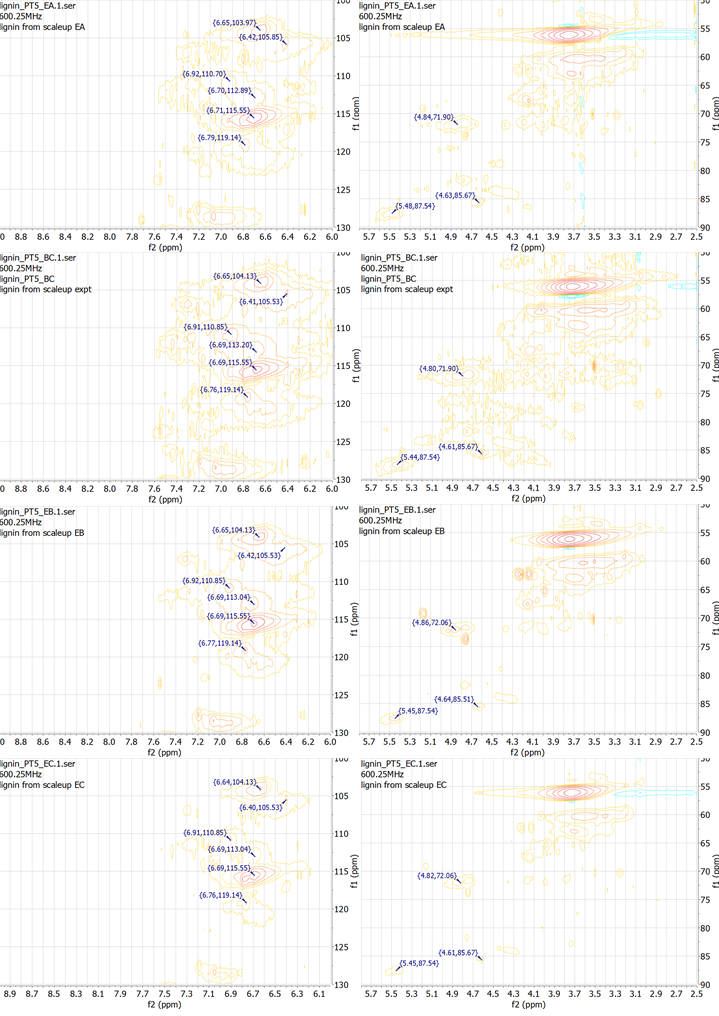


Figure ESI-5. HSQC spectra of the aromatic (left) and side chain (right) regions of IonoSolv lignins of *Miscanthus* with different particle sizes at 150 rpm. From top to botton: fine 10 wt% loading, fine 20 wt% loading, medium 10 wt% loading, coarse 10 wt% loading.


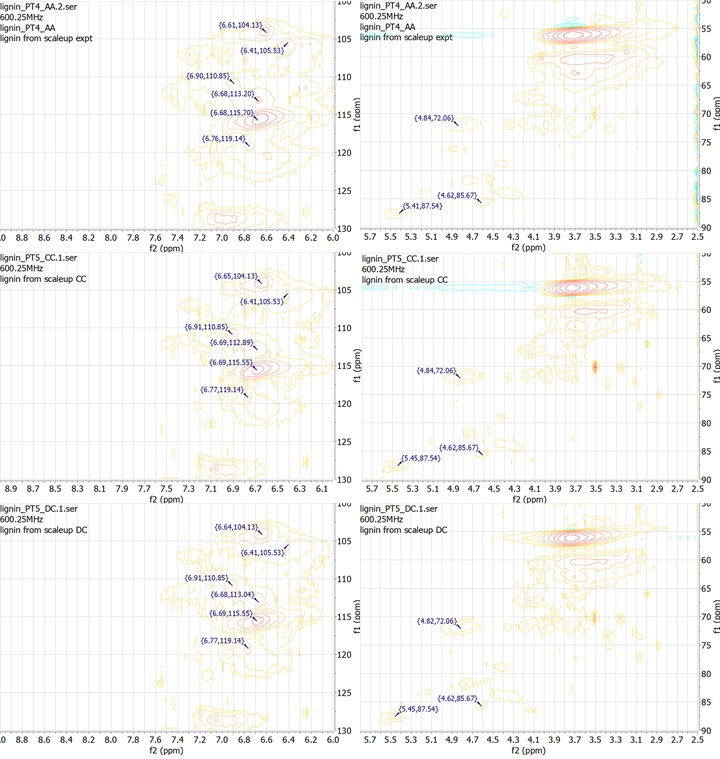


Figure ESI-6. HSQC spectra of the aromatic (left) and side chain (right) regions of IonoSolv lignins of *Miscanthus* with different particle sizes at 10 wt% loading and maximum power stirring. From top to botton: fine at 450 rpm, medium at 250 rpm, coarse at 400 rpm.

#### GPC

GPC measurements were performed using an Agilent 1260 Infinity instrument equipped with a Viscotek column set (AGuard, A6000M and A3000M). The Agilent 1260 Infinity RID detector was used for detection. HPLC grade DMSO containing LiBr (1 g/L) was used as eluent at a flow rate of 0.4 mL/min at 60 °C. Samples were prepared by dissolving 20 mg lignin in 1 ml eluent and filtering through a 0.2 µm syringe filter. Ten pullulan standards (Agilent calibration kit, 180 < M_p_ < 780,000) were used to calibrate the instrument.

Figure ESI-7. (a) Signal intensities in HSQC NMR relative to 100 G_2_+G_2,cond_ units and (b) molecular markers obtained by DMSO-eluted GPC for lignins recovered after pretreatment with [TEA][HSO_4_] as a function of biomass loading.

Figure ESI-8. HSQC NMR signal intensities of different lignin C-H units relative to 100 G_2_+G_2,cond_ units, lignin yield and delignification for lignins isolated from fine, medium and coarse *Miscanthus* particles pretreated at the 1-L scale using different mixing speeds.

Figure ESI-9. HSQC NMR signal intensities of different lignin C-H units relative to 100 G_2_+G_2,cond_ units , lignin yield and delignification for lignins isolated in scale-up lignin precipitates as a function of biomass loading and particle size.

### Thermal stability of [TEA][HSO_4_]

Thermogravimetric analysis of dried fresh IL (containing <0.5 wt% water as verified by Karl-Fischer titration) was performed on a TA Q500 (TA Instruments, USA) TGA analyzer fitted with a gas switching system, using a platinum pan, with N_2_ as the purge gas (20 mL/min). 20±5 mg of ionic liquid was loaded per run; each sample was analyzed in triplicate. The short-term stability of ILs was measured after a 30 min hold period at 80 °C, after which a temperature ramp (10 °C min^-1^) to 700 °C was performed. For determination of thermal stability, the onset of decomposition temperature (*T_onset_*) was determined. *T_onset_* is the intersection of the baseline weight and the tangent of the weight dependence on the temperature curve as decomposition occurs.

The thermal stability of dry [TEA][HSO_4_] was compared to [Emim][OAc] (Figure ESI-10), showing that [Emim][OAc] (T_onset_ of ~230 °C) is less thermally stable than [TEA][HSO_4_] (T_onset_ of ~ 275 °C).^12^ However, T_onset_ values are often considerably higher than the temperature at which the IL is stable for a long time period and therefore the maximum temperature where the IL can be used in a process without significant thermal weight loss.^12^

Figure ESI‑10. Thermogravimetric curves for [Emim][OAc] and [TEA][HSO_4_] upon temperature ramping to 700 °C in nitrogen atmosphere indicating the T_onset_ values.

A second isothermal experiment was conducted by placing ~1 g of fresh IL into a sealed glass vial and holding at 150 °C for 100 h. ^1^H NMR spectra were recorded for both dilute (20 wt% water) and neat fresh IL and after ageing at 150 °C for 100 hours continuously to observe the possible formation of by-products. This technique would flag levels of impurities greater than 1% relative to the IL. The spectrum with all peaks is shown in Figure ESI‑. Peaks that dominate the spectrum can be assigned to the cation, NMR solvent (DMSO-d_6_) and the protons on water/hydrogen sulfate. Significant signals for other proton-containing compounds were not discovered.

The long-term stability of [TEA][HSO_4_] upon constant heating in air was compared by ^1^H NMR spectroscopy before and after heating at 150 °C for 100 hours. Figure ESI-9 shows spectra that are visibly identical with no change in peak position or integrals after 100 h, indicating no chemical changes producing products above 1% concentration have taken place. The results indicate that decomposition reactions are undetectable or produce minimal levels of decomposition products at these temperatures, indicating these ILs are thermally stable at up to 150 °C.


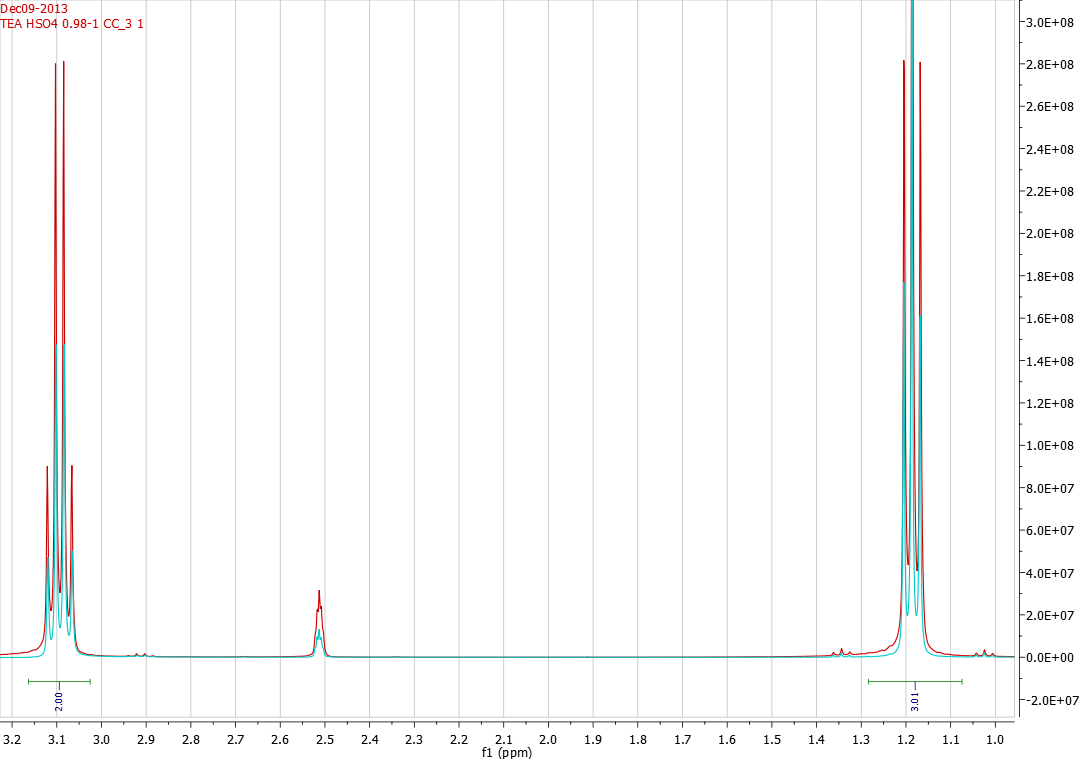


Before heat treatment

After heat treatment


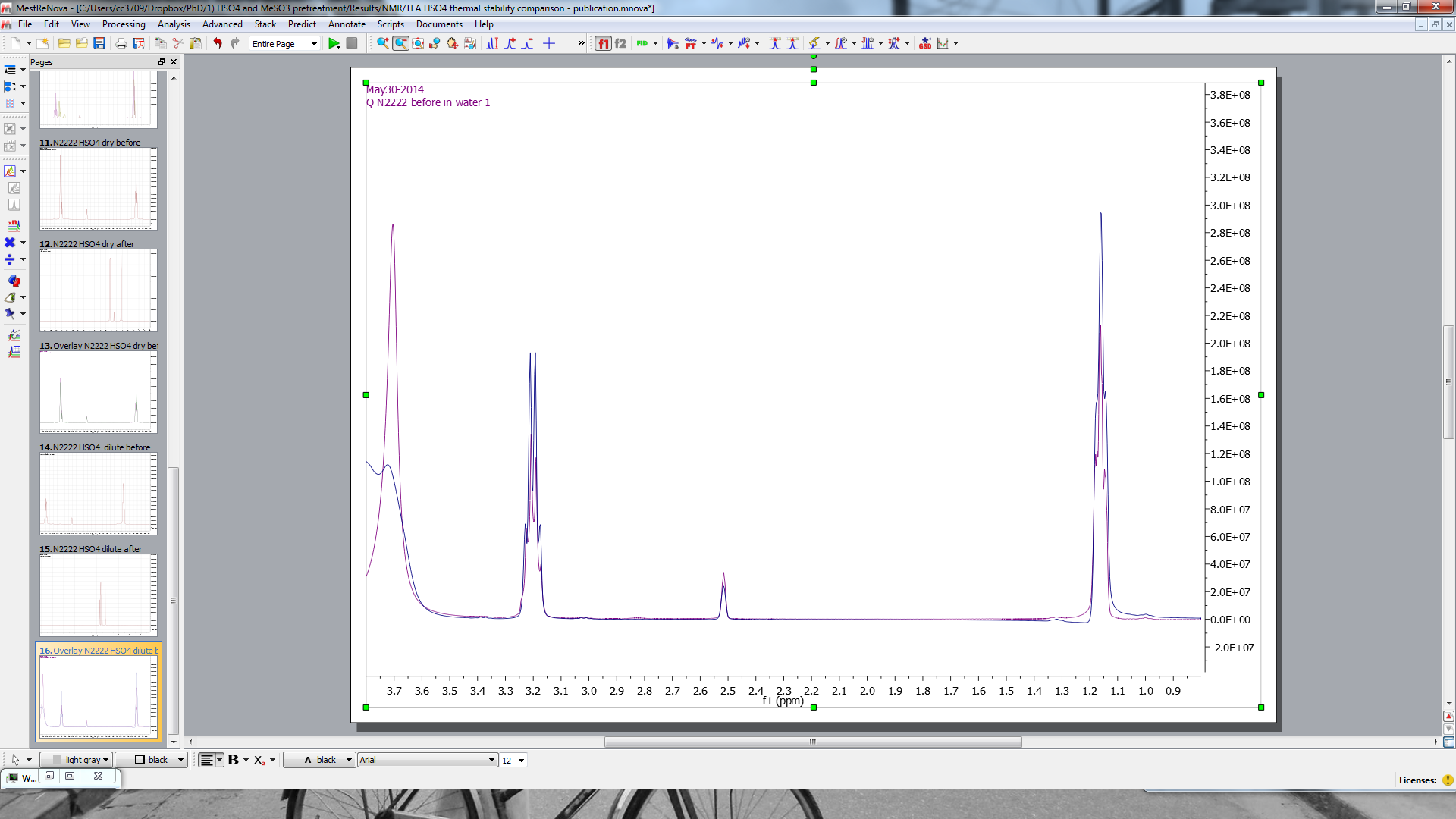


Before heat treatment

After heat treatment

Figure ESI‑11. ^1^H NMR spectrum (400 MHz; DMSO-d_6_) of [TEA][HSO4] with a water content of 20 wt% and acid:base ratio of 1:1 before (red) and after (blue) treatment at 150 °C for 100 hours. Shifts were reported in ppm with respect to TMS. Spectra shown are for (a) neat ionic liquid and (b) ionic liquid containing 20 wt% water.

### IL recovery quantification

The IL recovery was determined by comparing the quantity of IL recovered to the amount used during pretreatment (on a wt/wt dry basis, using Karl-Fischer titration).

For IL recovery quantification, the IL/water mixtures were concentrated by rotary evaporation to remove water and the residual weight and water content used to determine the recovery rate of the IL, *R* (wt%), given in Equation 1:

$R=\frac{{IL}_{rec}\times(100-w_{rec})}{{IL}_{fresh}\times(100-w_{fresh})}\times100$ (Equation 1)

where *IL_fresh_* is the total amount of IL added to the pretreatment tube and *w_fresh_*  is its water content while *IL_rec_* is the total amount of IL recovered and *w_rec_* is the water content of the recovered IL.

Figure ESI-12. IL recovery in the liquor relative to dry starting material.

### Ionic liquid solution acidity

The water content of fresh and recovered IL liquors were determined in triplicate by Karl Fisher titration (G20S compact titrator, Mettler Toledo). 0.1 g of IL (on a dry basis) was added into a 10 mL volumetric flask and mixed with deionized water (up to 10 mL). The solution was poured into a 10 mL Falcon tube and a calibrated pH meter (Mettler Toledo SevenEasy) used to measure the pH. The pH of the solution was converted into a proton concentration using Equation 14:

${{[H}^{+}]}_{IL}=\frac{{10}^{-pH}}{{IL}_{conc}}$ (Equation 14)

where [H^+^]_IL_ is the calculated proton concentration in the ionic liquid; pH is the measured pH value, IL_conc_ is the IL concentration in the analyzed sample (here 10 mg mL^-1^).

Table ESI‑3. Change of IL solution acidity with biomass loading. Miscanthus was pretreated at a biomass loading between 10 and 50 wt% in 80% [TEA][HSO_4_] with 20 wt% at 120 °C for 6 hours.

| **Sample** | **pH of 1 wt% solution^a^** | **[H^+^] concentration in IL (mmol/g) ^b^** | **H^+^ consumed with respect to fresh IL (%)** |
| --- | --- | --- | --- |
| Fresh TEA HSO_4_ | 1.65±0.01 | 0.224±0.001 | - |
| 10 wt% loading | 1.70±0.01 | 0.201±0.001 | 11 |
| 20 wt% loading | 1.73±0.02 | 0.188±0.002 | 19 |
| 30 wt% loading | 1.73±0.02 | 0.185±0.002 | 21 |
| 50 wt% loading | 1.75±0.01 | 0.178±0.001 | 26 |

^a^ Diluted 1:100 wt:wt in water on a dry basis. ^b^ calculated as H^+^ concentration in undiluted IL on a dry basis.

Figure ESI-13. pH of a 1% solution of IL liquor obtained after pretreatment with [TEA][HSO_4_] at biomass loadings from 2 to 50 wt%.

### Pulp washing optimization


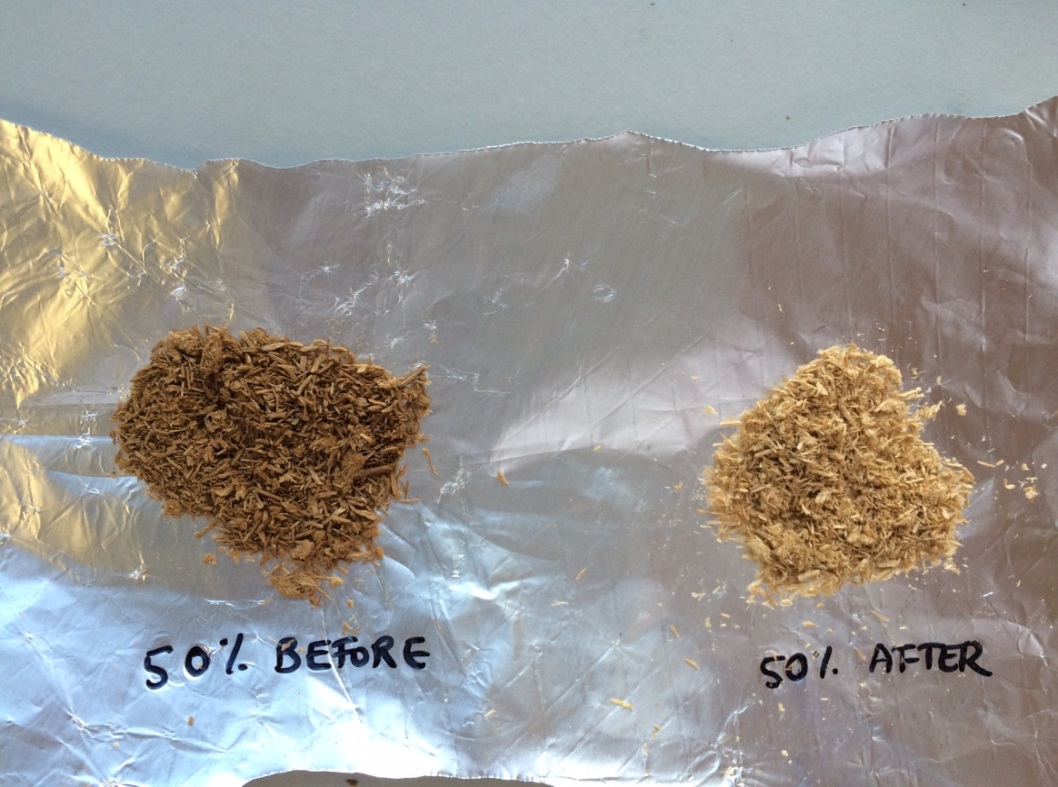

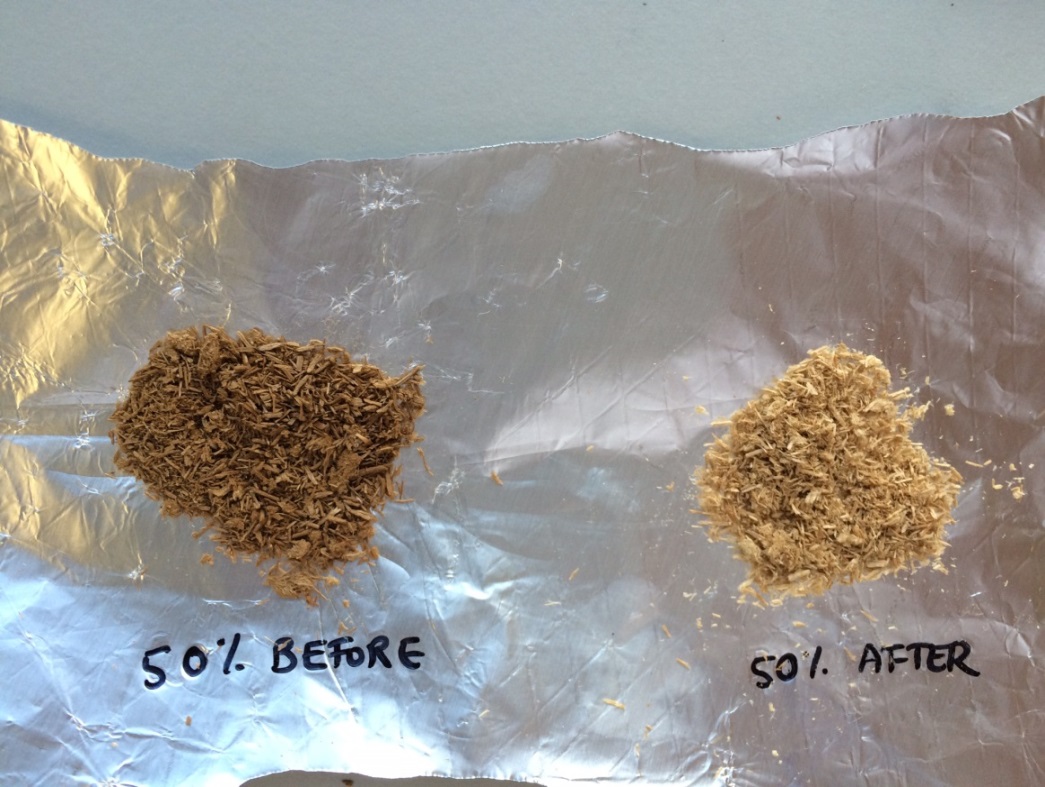

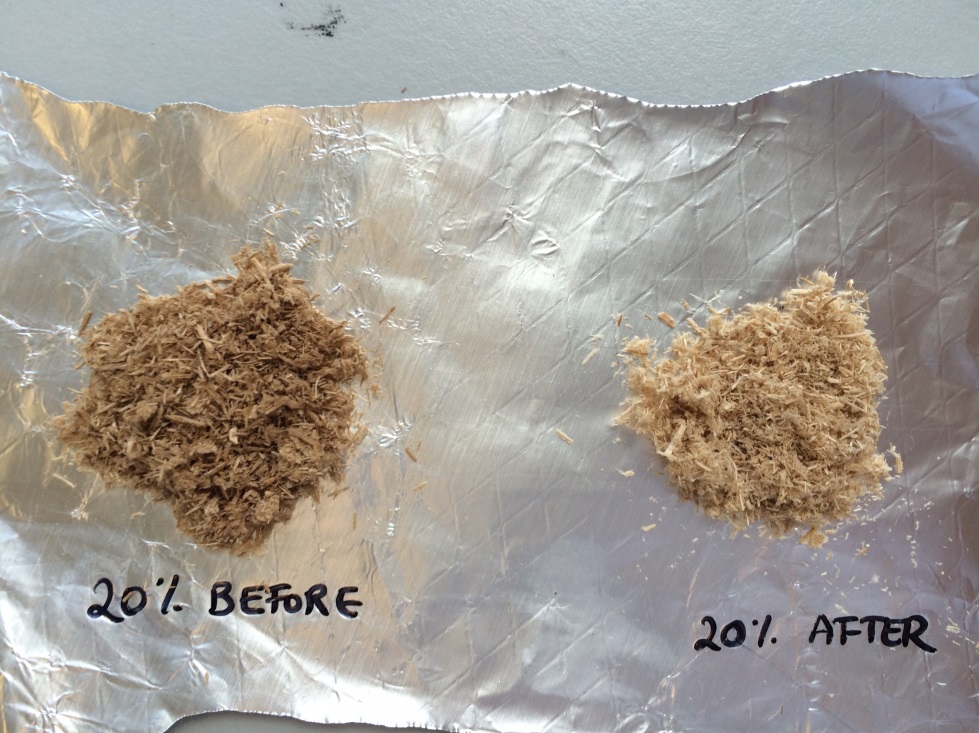

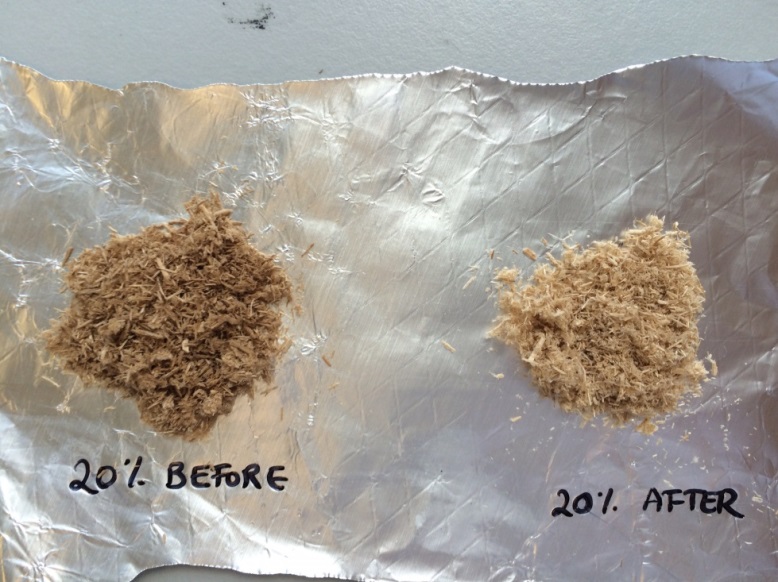


Figure ESI-14. Pretreated *Miscanthus* pulps obtained before and after washing with DMSO for biomass loadings of 20 wt% and 50 wt%.

Figure ESI-15. Amount of IL present and glucose yield obtained from pulp as a function of ethanol washes. Glucose yields shown as a percentage of the maximum glucose yield attained by vacuum filtered pulp after 16 equivalents.

Muslin cloth


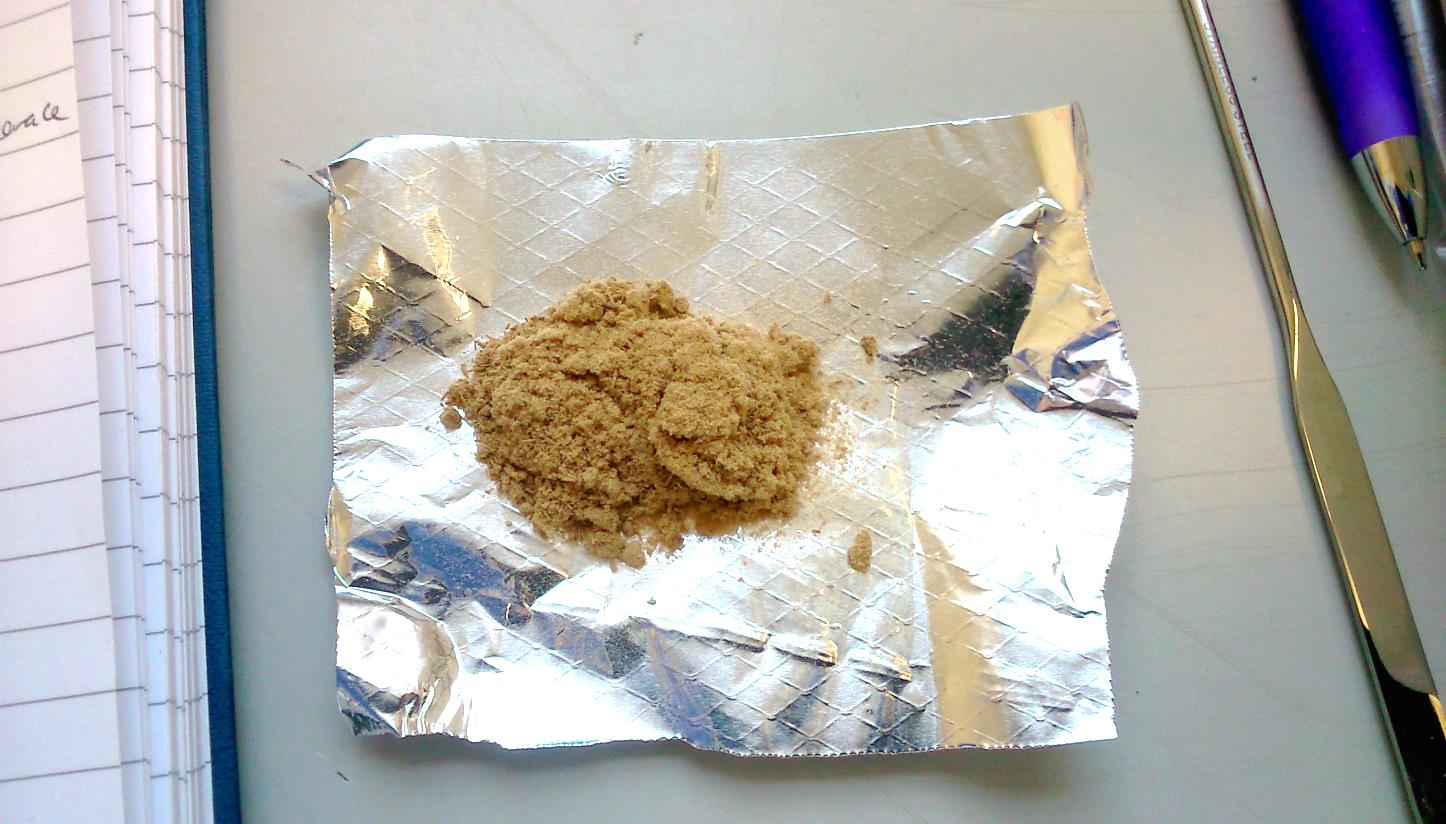

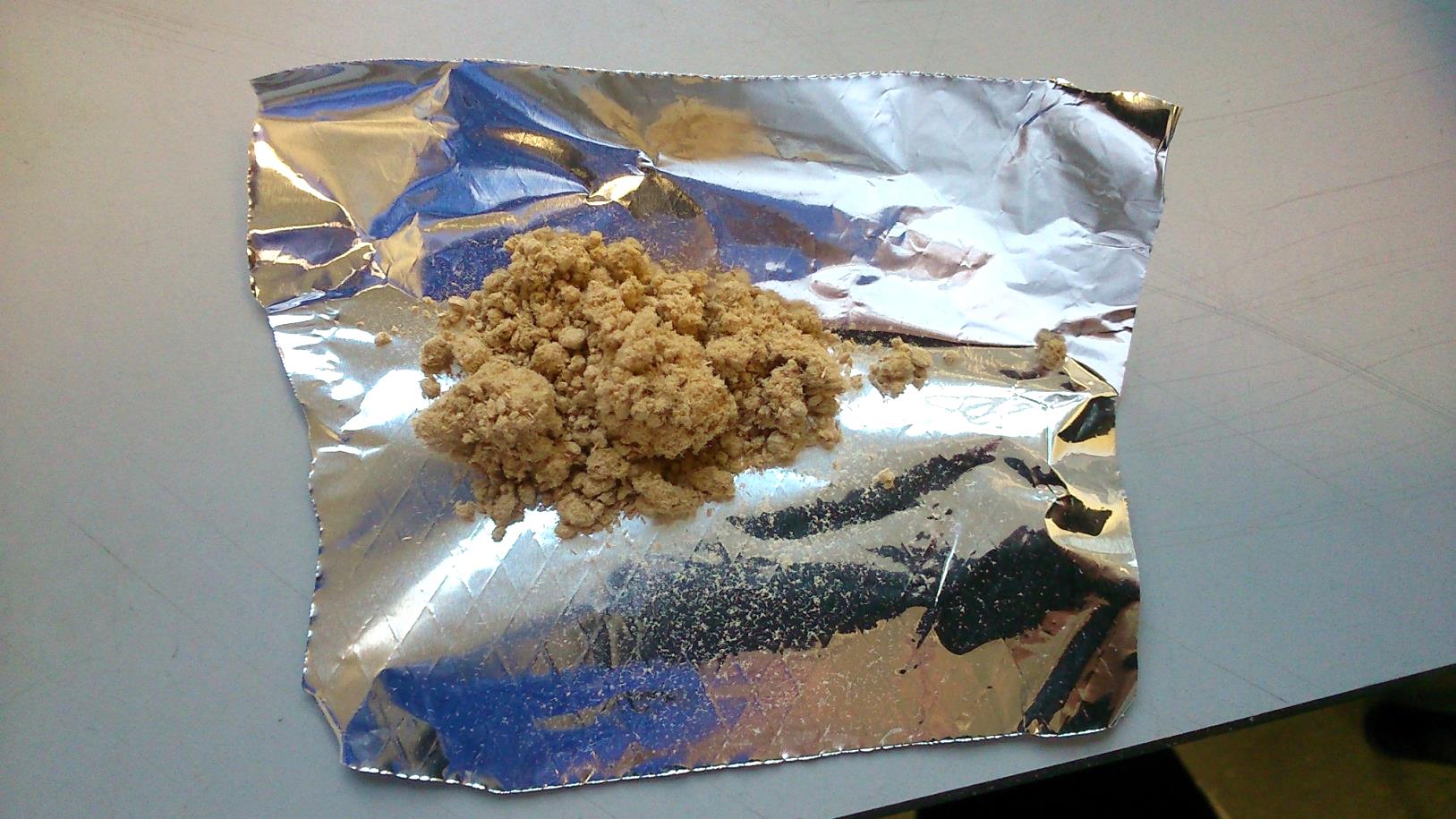

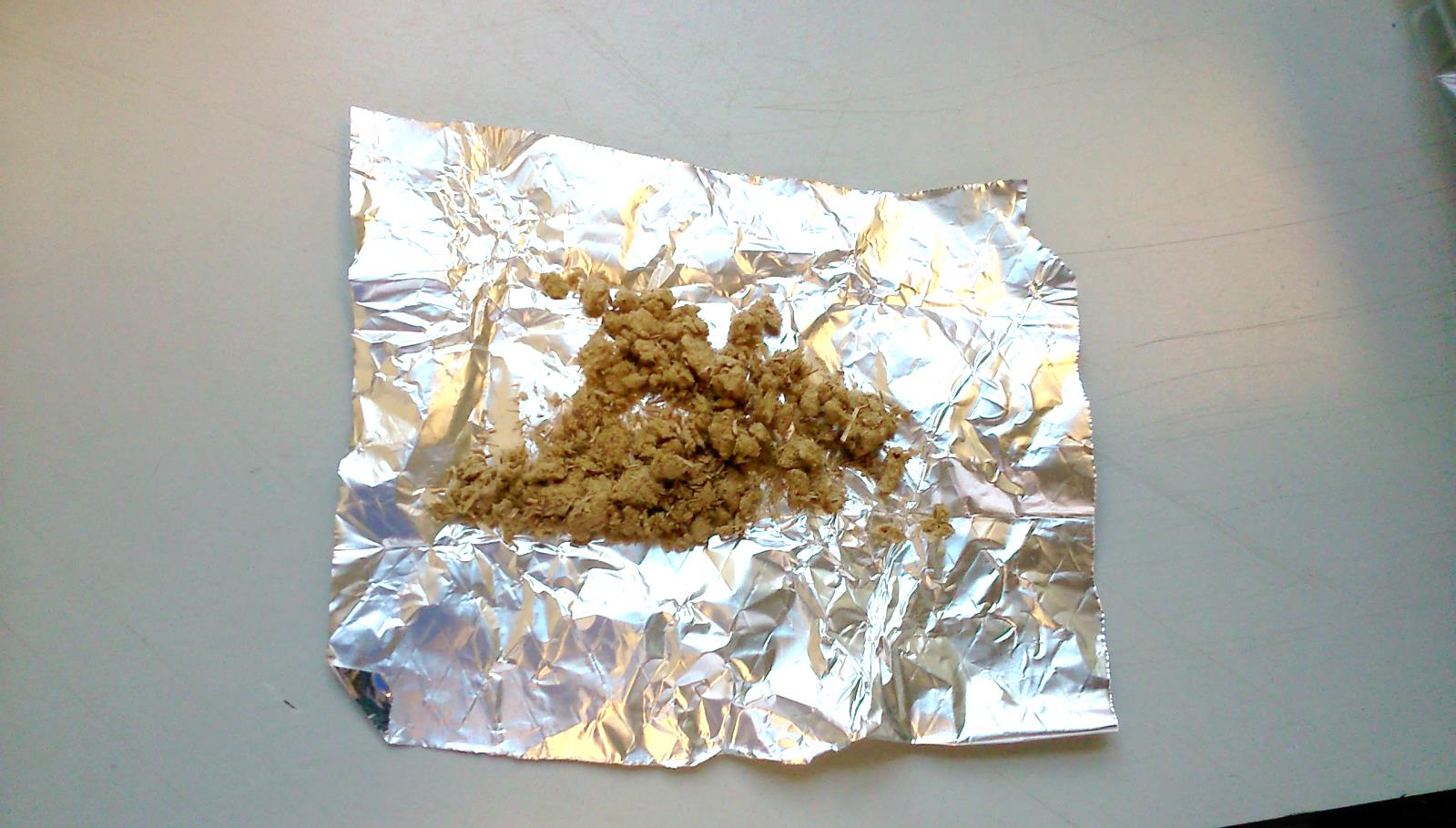


Centrifugation

Vacuum filtration

Figure ESI-16. Images of clean pulp after washing using 16 equivalents of ethanol by a) muslin cloth, b) centrifugation and c) vacuum filtration. Miscanthus pulp recovered after pretreatment of Miscanthus at 120 °C for 6 hours at 0.5L scale using stirring at 20 wt% loading.

### Effect of hornification

Figure ESI-17. Glucose yields obtained for air-dried and non-air-dried pulps after both EtOH washing and DMSO washing of pretreated Miscanthus pulps at 50 wt% biomass loading. NB. These experiments were conducted for a using a new batch of *Miscanthus* and IL; hence glucose yields for air-dried pulps are different to those presented above. These results are consistent within the batch and were obtained in a single saccharification experiment.

### Particle size distribution measurements

Particle size distributions were determined for untreated and pretreated pulps for all particle size experiments using nested sieves. The sample particle size distribution was obtained using a vibratory shaker (Retsch model SM 200, Hann, Germany) equipped with stacked sieves with decreasing pore sizes (5000, 4000, 3150, 2000, 1800, 1400, 1000, 850, 710, 500, 300, 212, 180, 150, 106, 75 and 53 μm) by shaking on a vibratory sieve shaker for 20 minutes for each sample.

The percentage weight of material retained by each sieve was measured and the log-normal distribution mass median diameter (*D_50_*), used as a measure of the average particle size of a biomass sample (i.e. the particle size that 50% of the sample is smaller than by mass when a log-normal distribution is fitted to the measurements), was calculated using Equation 8:

$D_{50}=10 ^ \left( \left( {\log x}_{2}- {\log x}_{1} \right) x \left( \frac{50- y_{1}}{y_{2}-y_{1}} \right)+\log x_{1} \right)$ (Equation 8)

where x_1_ and x_2_ are the pore sizes of the sieves (in mm) which allowed just under and just over 50% of the sample to pass through by weight, respectively, and y_1_ and y_2_ are the percentages of material passing through the sieves.

Particle size analyses were conducted in triplicates and percentage errors were assigned according to previous analysis for *Miscanthus* pulp particles, which found an average of 1% error for untreated biomass samples. Particle size distributions were plotted as weight percentages of samples passing through different sieve pore sizes (on a log scale) and geometric mean diameters *D_50_* were compared.

Figure ESI-18. Particle size distribution plotted as cumulative weight fraction of biomass particles passing through screens of successive sizes. Results shown for *Miscanthus* fine (above) and medium (below) size fractions before and after ionoSolv pretreatment. Inset values indicate percentage decrease in *D_50_* after treatment.

Figure ESI-19. Particle size distribution plotted as cumulative weight fraction of biomass particles passing through screens of successive sizes. Results are shown for *Miscanthus* treated by ionoSolv and dilute acid for fine (above) and medium (below) size fractions treated at 10 mL scale. Inset values indicate geometric mean particle size *D_50_*.

Figure ESI-20. Particle size distribution plotted as cumulative weight fraction of biomass particles passing through screens of successive sizes. Results are shown for fine (above), medium (middle) and coarse (below) size fractions of *Miscanthus* pretreated at different stirring speeds at 1 L scale. Inset values indicate geometric mean particle size *D_50_*.

### Dilute acid pretreatment

*Miscanthus* samples with the same three size fractions previously described were placed in pressurized glass pretreatment tubes. A solution of 3 wt% sulfuric acid in DI water (pH ~0.6) was added with dry solids loading of 1:5 g/g. The sealed glass tubes were then heated in a convection oven at 120 °C for 1.5 hours with three replicates. The acidic slurry was centrifuged, and 40 mL H_2_O added for washing. After pretreatment, the acidic slurry was transferred to 50 mL Falcon tubes for washing with DI water and separated by centrifugation (3000 rpm for 50 min or 2000xg). The supernatant was decanted and discarded and the washing step with DI water repeated three more times. The recovered solids were weighed to determine the pulp yield.

The combined severity factor (CSF) can be used to compare the pretreatment severity of dilute acid pretreatment processes, and is calculated using Equation 2^13^:

$CSF=log\left( t\times e\frac{T-100}{14.75} \right)-pH$ (Equation 1)

The conditions for dilute acid pretreatment were selected based on results from a previous study by Li *et al.*^14^, which used a CSF of ~2.0 (*T* of 160 °C, *t* of 15 min, [H_2_SO_4_] of 1.2 wt% i.e. pH of 0.93). Instead, we used conditions of *T* of 120 °C, *t* of 90 min, [H_2_SO_4_] of 3 wt% i.e. pH of 0.53) to maintain the same reaction temperature as used for ionoSolv experiments.

Figure ESI-21. Effect of particle size on *Miscanthus* key for dilute sulfuric acid pretreatment, after pretreatment with 3 wt% H_2_SO_4_ at 120 °C for 1.5 hours at bench scale using a biomass loading of 20 wt%.

#### Elemental analysis

Representative samples were selected from experiments conducted at 10 wt% and 50 wt% loadings. CHNS analysis of untreated air-dried biomass, pulp, lignin and recovered ionic liquid was performed in duplicate by MEDAC Ltd (Chobham, UK). Oxygen content was obtained by difference. Accuracy is ±0.30% absolute. CHNSO compositions and the calculated elemental composition of fresh ionic liquid were presented on a dry basis and used to conduct an elemental mass balance on the system (inputs: biomass and fresh IL in appropriate ratios; outputs: recovered IL, pulp and lignin in appropriate ratios) for both 10 wt% loading and 50 wt% loading.

### Mass balance

The elemental compositions of the inputs (untreated Miscanthus and fresh IL) and outputs (pretreated pulps, lignin and recovered IL) were used to determine the mass balance of the process at low and high solids loadings.

Table ESI-4. Elemental mass balance of the ionoSolv process conducted at bench scale for biomass loadings of 10 and 50 wt%.

|  | **10 wt% loading** | | **50 wt% loading** | |
| --- | --- | --- | --- | --- |
| **Mass in (g)** | | | | |
| Feedstock  C H N O S | 100.31  49.2  5.5  0.1  0.1  45.5 | | 100.31  49.2  5.5  0.1  0.1  45.5 | |
| Fresh ionic liquid (dry)  C H N O S | 801.07  289.7 68.9  56.3  128.9  257.3 | | 160.6  58.1  13.8  11.3  25.8  51.6 | |
| TOTAL IN  C  H  N  O  S | 901.37  338.9  74.4  56.4  129.0  302.8 | | 260.9  107.3  19.3  11.3  25.9  97.1 | |
| **Mass out (g)** | Actual | Corrected ^a^ | Actual | Corrected ^a^ |
| Pulp  C  H  N  O  S | 48.8  22.2  2.5  0.1  0.6  23.5 | - | 57.9  28.9  2.9  0.1  0.7  25.4 | - |
| Lignin  C  H  N  O  S | 21.7  13.5  1.3  0.2  0.4  6.4 | - | 13.6  8.5  0.8  0.1  0.2  4.1 | - |
| Ionic liquid liquor (dry)  C  H  N  O  S | 792.6  288.1  73.2  53.6  123.4  254.3 | 801.1  291.2  73.9  54.2  124.7  257.1 | 147.9  54.6  13.6  9.4  21.6  48.7 | 160.6  59.3  14.8  10.2  23.5  52.9 |
| TOTAL OUT  C  H  N  O  S | 863.1  323.8  76.9  53.9  124.4  284.2 | 871.6  326.9  77.7  54.4  125.7  286.9 | 219.5  92.0  17.4  9.5  22.5  78.2 | 232.2  96.7  18.5  10.3  24.3  82.4 |
| **Mass recovery (wt%)** | | | | |
| Total  Losses  C  H  N  O  S | 95.8  4.2  95.5  103.5  95.6  96.5  93.9 | 96.7  3.3  96.5  104.5  96.6  97.5  94.8 | 84.1  15.9  85.7  90.0  83.9  86.7  80.5 | 89.0  11.0  90.1  96.0  91.0  93.9  84.8 |

^a^ Calculations performed assuming 100 wt% IL recoveries (instead of 98.9 and 92.1 wt% for 10 and 50 wt% loading).

hours. Glucose yields shown relative to the amount of glucan in untreated biomass.

### Effect of stirring

Table ESI-5 Effect of mixing speed on key indicators of pretreatment and lignin characteristics from *Miscanthus*.

|  | **Stirring speed** | **Delignification (wt%)** | **Lignin yield (wt%)** | **Saccharifi-cation (%)** | **M_w_ (g/mol)** | **Ð (-)** | **S/G ratio (-)** | **β-*O*-4’ abundance** |
| --- | --- | --- | --- | --- | --- | --- | --- | --- |
| Fine | Low | 87 | 88.5 | 79.1 | 4900 | 4.1 | 0.70 | 20.3 |
|  | Fast | 88 | 88.8 | 66.1 | 4620 | 3.7 | 0.66 | 10.2 |
| Medium | Low | 83 | 76.8 | 68.2 | 5760 | 4.4 | 0.69 | 19.5 |
|  | Fast | 83 | 84.7 | 67.8 | 5720 | 5.4 | 0.71 | 16.5 |
| Coarse | Low | 69 | 63.4 | 59.3 | 6500 | 5.3 | 0.79 | 24.8 |
|  | Fast | 87 | 83.7 | 59.0 | 5610 | 4.7 | 0.74 | 17.4 |

### References

1 F. J. V Gschwend, A. Brandt, C. L. Chambon, W.-C. Tu, L. Weigand and J. P. Hallett, *JoVE*, 2016, e54246.

2 C. L. Chambon, V. Fitriyanti, P. Verdía, S. M. Yang, S. Hérou, M.-M. Titirici, A. Brandt-Talbot, P. S. Fennell and J. P. Hallett, *ACS Sustain. Chem. Eng.*, 2020, **8**, 3751–3761.

3 A. Procentese, E. Johnson, V. Orr, A. [Garruto Campanile], J. A. Wood, A. Marzocchella and L. Rehmann, *Bioresour. Technol.*, 2015, **192**, 31–36.

4 A. Sluiter, B. Hames, R. Ruiz, C. Scarlata, J. Sluiter, D. Templeton and D. Crocker, *NREL/TP-510-42618, Determination of Structural Carbohydrates and Lignin in Biomass: Laboratory Analytical Procedure (LAP)*, 2008.

5 M. G. Resch, J. O. Baker and S. R. D. Nrel, *Low Solids Enzymatic Saccharification of Lignocellulosic Biomass Low Solids Enzymatic Saccharification of Lignocellulosic Biomass Laboratory Analytical Procedure ( LAP )*, 2015.

6 M. Sette, R. Wechselberger and C. Crestini, *Chem. - A Eur. J.*, 2011, **17**, 9529–9535.

7 J. X. Sun, X. F. Sun, R. C. Sun, P. Fowler and M. S. Baird, *J. Agric. Food Chem.*, 2003, **51**, 6719–6725.

8 A. Brandt, F. Gschwend, P. Fennell, T. Lammens, B. Tan, J. Weale and J. Hallett, *Green Chem.*, 2017, 3078–3102.

9 C. Crestini and D. S. Argyropoulos, *J. Agric. Food Chem.*, 1997, **45**, 1212–1219.

10 W. Lan, C. Liu and R. Sun, *J. Agric. Food Chem.*, 2011, **59**, 8691–8701.

11 G. Hu, C. Cateto, Y. Pu, R. Samuel and A. J. Ragauskas, *Energy & Fuels*, 2012, **26**, 740–745.

12 Y. Cao and T. Mu, *Ind. Eng. Chem. Res.*, 2014, **53**, 8651–8664.

13 J. Shekiro, E. M. Kuhn, N. J. Nagle, M. P. Tucker, R. T. Elander and D. J. Schell, *Biotechnol. Biofuels*, 2014, **7**, 1–10.

14 C. Li, L. Sun, B. A. Simmons and S. Singh, *Bioenergy Res.*, 2013, **6**, 14–23.
